# The global epidemiology and treatment of metallo-β-lactamase-producing gram-negative pathogens

**DOI:** 10.1093/jacamr/dlag221

**Published:** 2026-10-14

**Authors:** Erin K McCreary, Emily L Heil, Samuel L Aitken, Maria F Mojica, Jason M Pogue, Alejandro J Vila, Ryan K Shields

**Affiliations:** Division of Infectious Diseases, Department of Medicine, University of Pittsburgh, Pittsburgh, PA, USA; Department of Practice, Sciences, and Health-Outcomes Research, University of Maryland School of Pharmacy, Baltimore, MD 21201, USA; Department of Clinical Pharmacy, University of Michigan College of Pharmacy, Ann Arbor, MI, USA; Department of Pharmacy, Michigan Medicine, Ann Arbor, MI, USA; Division of Infectious Diseases, University of Michigan Medical School, Ann Arbor, MI, USA; Department of Molecular Biology and Microbiology, Case Western Reserve University School of Medicine, 9501 Euclid Ave, Cleveland, OH 44106, USA; Research Service, Veterans Affairs Northeast Ohio Healthcare System, Cleveland, OH, USA; CASE-VA Center for Antimicrobial Resistance and Epidemiology, Cleveland, OH, USA; Department of Clinical Pharmacy, University of Michigan College of Pharmacy, Ann Arbor, MI, USA; Department of Pharmacy, Michigan Medicine, Ann Arbor, MI, USA; Instituto de Biología Molecular y Celular de Rosario (IBR), CONICET, Universidad Nacional de Rosario, Ocampo & Esmeralda, Rosario S2000EXA, Argentina; Facultad de Ciencias Bioquímicas y Farmacéuticas, Universidad Nacional de Rosario, Rosario, Argentina; Division of Infectious Diseases, Department of Medicine, University of Pittsburgh, Pittsburgh, PA, USA

## Abstract

Infections caused by metallo-β-lactamase (MBL)-producing gram-negative pathogens represent a formidable and emerging threat to public health. MBLs compromise the activity of carbapenems and many newly developed β-lactam/β-lactamase inhibitor agents, leaving few treatment options. Remarkably, these enzymes have disseminated globally at an unprecedented rate. Therapeutic management of MBL-producing pathogens remains a major challenge because the enzymes are not inhibited by currently available β-lactamase inhibitors, and most pathogens that harbour MBLs are equipped with mechanisms of resistance to all available antibiotic classes. Recent advances have elucidated the biochemical and structural function of diverse MBLs thereby improving the development of future antibiotics. In this review, we summarize the contemporary global epidemiology of clinically relevant MBLs, describe the molecular mechanisms driving their dissemination and evaluate current and emerging therapeutic strategies for infections caused by MBL-producing gram-negative pathogens including Enterobacterales, *Pseudomonas aeruginosa, Acinetobacter baumannii* and *Stenotrophomonas maltophilia*.

## Introduction

Metallo-β-lactamases (MBLs) are a highly diverse group of Ambler Class B enzymes that hydrolyse β-lactam antibiotics via a zinc-mediated catalytic mechanism. They account for approximately 10% of all known β-lactamase enzymes and are divided into subclasses B1, B2 and B3 based on phylogeny, active-site architecture, zinc coordination and substrate profiles.^1^ Enzymes in the B1 and B3 subfamily coordinate two Zn(II) ions and have broad-spectrum hydrolytic activity against penicillins, cephalosporins and carbapenems, whereas B2 enzymes are active with one Zn(II) ion at the active site and their spectrum is limited to carbapenems. Neither B1, B2 nor B3 enzymes hydrolyse aztreonam (ATM).

Mechanistically, MBLs differ from serine β-lactamases (i.e. Ambler Classes A, C and D) in that serine enzymes rely on an active-site serine that attacks the β-lactam carbonyl, forming a high-energy tetrahedral intermediate that collapses to form a covalent acyl-enzyme intermediate.^2^ In contrast, MBLs activate a Zn-bound hydroxide ion that directly attacks the β-lactam carbonyl, forming a transitional high-energy tetrahedral intermediate that collapses into an anionic intermediate upon β-lactam ring opening. In both systems, a water molecule mediates product release, but the absence of a covalent intermediate in MBLs underlies their resistance to classical β-lactamase inhibitors.^1^ This mechanism may confer greater *in vitro* resistance than *in vivo* resistance given that antimicrobial susceptibility testing is performed in zinc-rich media and infection generally occurs in a state of zinc deprivation that negatively impacts hydrolytic activity, protein folding and ultimately patient outcomes.^3^ Zinc deprivation has been shown to induce MBL degradation in different bacterial hosts, but its link to resistance is unclear and remains a subject of debate.^4–6^

MBLs are widely distributed among gram-negative pathogens and the only β-lactamase family found in all three domains of life.^7^ Clinically significant plasmid-borne B1 enzymes include Imipenemase (IMP), Verona integron-encoded (VIM), New Delhi (NDM) and São Paulo. Other B1 MBLs are chromosomally encoded, such as IND in *Chryseobacterium indologenes,* BlaB in *Elizabethkingia meningoseptica,* MUS/MYO in *Myroides odoratimimus* and CfiA in *Bacteroides fragilis. Chryseobacterium* spp. have been suggested to act as environmental reservoirs of MBL genes, particularly NDM-1 that is bound to cell membranes.^8^ Subclasses B2 and B3 are chromosomal MBLs identified in *Aeromonas spp.* (CphA, B2), *Serratia fonticola* (Shf-1, B2), *Stenotrophomonas maltophilia* (L1, B3) and *E. meningoseptica* (GOB, B3).^9^ Treatment of pathogens with plasmid-borne B1 enzymes is often complicated by other antibiotic resistance genes co-harboured on plasmids. Moreover, the extreme sequence diversity within Class B enzymes provides significant challenges to broad-spectrum β-lactamase inhibitor development leaving few therapeutic options.^1^

Infections caused by MBL-producing bacteria are increasing globally at a rapid rate. From 2019 to 2021, amongst 261 consecutive clinical carbapenem-resistant Enterobacterales (CRE) isolates in US hospitals, the percentage harbouring MBLs rose from 4% to 20%.^10^ Unfortunately, antimicrobial countermeasures have lagged behind the emergence of MBLs as there are currently no commercially available MBL inhibitors.^11^ This narrative review summarizes global epidemiology, delineates risk factors and clinical outcomes and focuses on the role of new and emerging β-lactam and β-lactamase inhibitor-based therapeutic strategies for infections caused by MBL-producing organisms (Table 1).

**Table 1.** Considerations for treatment of MBL-producing Enterobacterales and *P. aeruginosa*

Pathogen	MBL type	Preferred empiric treatment if enzyme and organism are known	Reasonable alternatives(s)	Considerations
*E. coli*	NDM	ATM-AVI FEP-ZID ATM PLUS SUL-DUR ATM PLUS FEP-ZID	Ceftazidime/avibactam plus ATM	The authors avoid cefiderocol for NDM-harbouring Enterobacterales due to elevated MICs, ability to be hydrolysed and suboptimal outcomes in clinical trials. Multiple options are listed for NDM *E. coli* due to rapid global expansion of isolates harbouring PBP3 insertions and/or CMY- or CTX-M enzymes. FEP-ZID may have more potent *in vitro* activity against these isolates compared to ATM-AVI; however, time-kill assays show similar antibacterial activity of ATM-AVI and FEP-ZID against these mutants. There are no comparative clinical data and extremely limited clinical data for all available antibiotics. ATM plus zidebactam or ATM plus durlobactam may be *in vitro* active and have demonstrated potency against isolates with PBP3 insertions.
Other Enterobacterales	NDM	ATM-AVI	Ceftazidime/avibactam plus ATM FEP-ZID	
	IMP, VIM	ATM-AVI	Ceftazidime/avibactam plus ATM FEP-ZID Cefiderocol	
*P. aeruginosa*	NDM	FEP-ZID	Cefiderocol, in combination with other *in vitro* active agent Polymyxins	
	IMP, VIM	Cefiderocol (+/− another *in vitro* active agent)	FEP-ZID Polymyxins	

Treatment recommendations are in the context of assuming all drugs are available at the time of decision-making. Other considerations, assuming the antibiotic is *in vitro* susceptible, are as follows: (i) ATM, ciprofloxacin, levofloxacin or TMP/SMX are reasonable for MBL-producing infections; (ii) tigecycline or eravacycline are reasonable for intra-abdominal, skin and soft tissue or bone/joint infections due to Enterobacterales; (iii) nitrofurantoin or oral fosfomycin are reasonable for uncomplicated urinary tract infections due to *E. coli*; (iv) aminoglycosides are reasonable for urinary tract infections due to Enterobacterales or *P. aeruginosa*; (v) antimicrobial susceptibility testing should always be obtained for any antibiotic being used for definitive treatment.

## History and enzymology of MBLs

MBLs belong to the metallo-hydrolase/oxidoreductase superfamily of enzymes. Despite sharing <5% amino acid identity across the broader superfamily, all members share a conserved structural scaffold known as the ‘MBL fold’, an αββα-fold in which the active site lies at the edge of the central β-sheets.^12^ This fold supports a metal-binding motif that coordinates the metal ions, but the precise arrangement of active-site residues varies substantially among enzymes.

The first zinc-dependent β-lactamase activity was described in 1966, when Sabath and Abraham^13^ noted an increase in cephalosporinase activity in a crude enzyme extracted from *Bacillus cereus* after the addition of zinc, and that this activity was abolished by ethylenediaminetetraacetic acid (EDTA). Whether zinc activity was ‘peculiar’ to *B. cereus* cephalosporinases was unknown at the time, but the authors recognized that this enzyme did not resemble previously characterized β-lactamases. Subsequent biochemical studies on this enzyme—later designated BcII—became foundational to the field and provided the mechanistic framework that was revisited decades later as MBLs emerged as major clinical threats.^14–18^ Notably, BcII acts as a carbapenemase despite being discovered decades before clinical use of carbapenems.^19^

The B1 enzymes IMP, VIM and NDM are the focus of this review. The canonical active site of B1 MBLs contains two metal-binding sites, known as the Zn1 and Zn2 sites. The Zn1 ion is coordinated to residues His116, His118 and His196 (the 3H site), whereas Zn2 is coordinated by Asp120, Cys221, His263 (the DCH site) and a water molecule. A bridging hydroxide links the two metals and serves as the activated nucleophile for β-lactam hydrolysis. Residues that interact with the metal-coordinating residues through hydrogen bonds or salt bridges are referred to as second-shell residues.^1^ Substitutions in the primary coordinating residues typically impair both catalytic efficiency and zinc binding, explaining their high degree of conservation across B1 enzymes.^20–23^ Lastly, the B1 active site forms a shallow groove bordered by two flexible loops, L3 and L10 (corresponding to BBL positions ∼59–67 and 223–241, respectively). This architecture allows accommodation of penicillins, cephalosporins and carbapenems, but not ATM. While two residues in L10—Lys224 and Asn233—are highly conserved, other positions within L3 and L10 vary across IMP, VIM and NDM enzymes. These substitutions fine-tune substrate affinity and catalytic preferences without altering the overall broad-spectrum profile. As a result, NDM, VIM and IMP enzymes generally hydrolyse most β-lactam substrates with comparable efficiency, though some variants hydrolyse certain molecules more efficiently than others.^1^

IMP was first identified in 1988 from a clinical *Pseudomonas aeruginosa* isolate in Japan by Watanabe *et al.*^24^ who reported imipenem inactivation that was inhibited by EDTA and partially restored by divalent cations. This was the first reported zinc-dependent carbapenemase in a clinically relevant gram-negative pathogen that was also able to transfer by conjugation to other *P. aeruginosa* strains. The enzyme was formally designated IMP when Osano *et al.*^25^ characterized IMP-1 from a *Serratia marcescens* clinical isolate in 1994. Sequence analysis revealed homology to previously reported MBLs from *B. cereus, Bact. fragilis* and *Aeromonas hydrophila,* and biochemical analysis confirmed broad hydrolytic activity against all β-lactams except ATM and inhibition by EDTA. Interestingly, IMP-1 was chromosomally encoded, and although conjugation into *Escherichia coli* was not observed in the initial report, the authors recognized the potential for horizontal exchange among Enterobacterales. Now, several years later, it is well-established that *bla*_IMP_-like genes are mobilized as gene cassettes within Class 1 and Class 3 integrons that enable efficient dissemination across diverse gram-negative species. Indeed, in Australia, an IMP-4 enzyme propagated from *Se. marcescens* to *P. aeruginosa* and to multiple other bacterial species through plasmid transmission mobilized by transposons of the Class 1 integrons and clonal propagators.^26^

As of 2026, 111 IMP-like MBLs have been annotated in NCBI (2.9 variants/year), which demonstrate significant diversity in an unrooted phylogenetic tree. The most common variants are IMP-1, IMP-4 and IMP-8.^27,28^ A defining biochemical feature of IMP enzymes is their high zinc affinity, which enhances resistance to chelating agents and helps maintain catalytic activity under zinc-limited conditions.^29^ Biochemical studies have demonstrated carbapenem use as a driver of IMP variant evolution.^30,31^ IMP-like enzymes are generally more resistant to inhibition by β-lactamase inhibitors in development, such as taniborbactam and xeruborbactam, when compared to other MBLs.

VIM-1 was first reported in 1999, and as of 2026, 96 annotated variants have been reported (3.5 variants/year).^27,32^ These enzymes fall into three major phylogenetic groups: VIM-1-related, VIM-2-related and VIM-7-related. The first VIM-1 enzyme was identified in *P. aeruginosa* (VR-143/97) from an inpatient at the Verona University Hospital in Italy.^32^  *bla*_VIM_ was located on the chromosome within a Class 1 integron gene cassette. Although not transferable by conjugation to *E. coli*, the authors predicted that due to the mobile nature of gene cassettes, *bla*_VIM_ will transfer to conjugative plasmids—which alas, has been proven true. However, while VIM-1 has disseminated into many gram-negative species, the *bla*_VIM-2_ gene is rarely found outside of *P. aeruginosa.* This restriction is driven by the leader peptide of VIM-2, which imposes a fitness cost in other hosts by impairing efficient translocation and processing of the enzyme. As a result, VIM-2 expression is toxic to *E. coli* and *Klebsiella* spp., thereby preventing species transmission.^27^

VIM enzymes have a broad substrate profile including penicillins, cephalosporins and carbapenems; however, like other MBLs, they do not hydrolyse ATM.^32^ Some variants exhibit higher catalytic efficiency for certain cephalosporins. For example, cefotaxime has a higher turnover rate than ceftazidime or cefepime. VIM-7 exhibits lower cephalosporinase activity than other variants. In general, VIM enzymes have a higher catalytic efficiency for imipenem than meropenem and for meropenem more than ertapenem.^27^ Variations in catalytic activity for both VIM and IMP variants are mediated by mutations in loops, substrate binding residues and second sphere residues.

Yong *et al.*^33^ first reported NDM-1 in 2009 from a carbapenem-resistant *Klebsiella pneumoniae* isolate recovered in Sweden from a patient recently hospitalized in New Delhi, India. Whole-genome sequencing revealed three distinct resistance regions, including two novel determinants: *bla*_NDM-1_ and an erythromycin esterase gene. Subsequent sampling identified NDM-1 in *E. coli* from the patient’s stool, suggesting *in vivo* plasmid-mediated transfer. NDM-1 shares limited identity with other MBLs; the closest homologues, VIM-1 and VIM-2, exhibit only 32.4% sequence identity. Moreover, NDM are unique among MBLs in that they are outer-membrane-anchored lipoproteins that can be packaged into outer membrane vesicles in *E. coli*, *P. aeruginosa* and *Acinetobacter baumannii* without imposing a fitness cost. These factors have contributed to their rapid global dissemination.^4,34^ This cellular localization also prevents NDM degradation in the periplasm under zinc-limiting conditions.^4^

As of 2026, 94 NDM-like variants are annotated in NCBI, representing the fastest rate of diversification among MBL families (5.5 variants/year). As with IMP- and VIM-like enzymes, NDMs can hydrolyse all β-lactams except ATM, with subtle kinetics differences. For instance, NDM-like enzymes demonstrate greater binding affinity to penicillins and cephalosporins (e.g. cefuroxime, cefotaxime) compared to VIM-2 and lower binding affinity to carbapenems than VIM-2 or IMP-1, but a similar rate of carbapenem turnover as VIM-2.^33^ Currently, NDM-1 exhibits the slowest steady-state kinetic hydrolysis of meropenem and imipenem compared to NDM-5 (fastest, with greatest hydrolytic activity against meropenem) and NDM-7 (second fastest).^35^ However, NDM-like enzymes are highly conserved, with an average amino acid identity of 94% and typically only 1–2 amino acid changes between variants, resulting in generally similar hydrolytic profiles. Therefore, substrate affinity is not the main driving force behind the evolution of NDM variants. Instead, multiple studies have shown NDM variants evolve to optimize resistance under Zn deprivation and to enhance their structural stability.^5,36–38^ A common variant that exhibits these features is NDM-5.^39^

NDM-1, NDM-5 and NDM-7 are the most prevalent globally disseminated variants. The *bla*_NDM_ gene is commonly linked to insertion sequence common region 1, a mobilization element that enables highly efficient movement between plasmids and chromosomes. This structure has been identified in over 20 different highly transmissible plasmids and is frequently co-located with additional resistance genes such as *sul*1 [conferring trimethoprim/sulfamethoxazole (TMP/SMX) resistance], *qnr*S (conferring fluoroquinolone resistance) and *rmt*B (conferring aminoglycoside resistance). Remarkably, *bla*_NDM_ has been detected in more than 40 bacterial species; however, transmission occurs most efficiently through *K. pneumoniae*.^40^ Across species, *bla*_NDM-1_ and *bla*_NDM-7_ are found more commonly in *K. pneumoniae*, whereas *bla*_NDM-5_ is more often identified in *E. coli.* Indeed, *bla*_NDM-5_ is carried on an F-type plasmid in two notable high-risk *E. coli* clones: ST167 and ST410.^41,42^ The most common NDM variant varies by geographic region, underscoring the importance of tracking local epidemiology.^43–45^

## Global emergence of MBL-producing gram-negative pathogens

Since their initial identification, the dissemination of MBLs has been marked by the rapid emergence in geographically diverse regions (Figure 1). Kanj *et al.*^46^ systematically reviewed reported cases of MBL-producing Enterobacterales from 2013 through 2023 and noted most IMP-like enzymes originated from Asia, while most VIM-producing isolates were reported from Europe, and NDM was the most diverse MBL identified across various regions. The current distribution of IMP-like enzymes (Figure 1a) still follows this epidemiologic trend, as IMP enzymes are endemic in Japan and China, the epicentre of IMP dissemination. Remarkably, IMP-18 and IMP-62 are the dominant MBLs in *P. aeruginosa* in Peru, making this South American country one of the few non-Asian countries where IMPs are highly prevalent. The prevalence of IMP enzymes in Australia, Brazil and much of Southern and Eastern Europe and Southeast Asia is considered moderate. North America, South America, Scandinavia and the Baltics are regions with low prevalence of IMP-like enzymes.^27,28,47–51^

**Figure 1.**
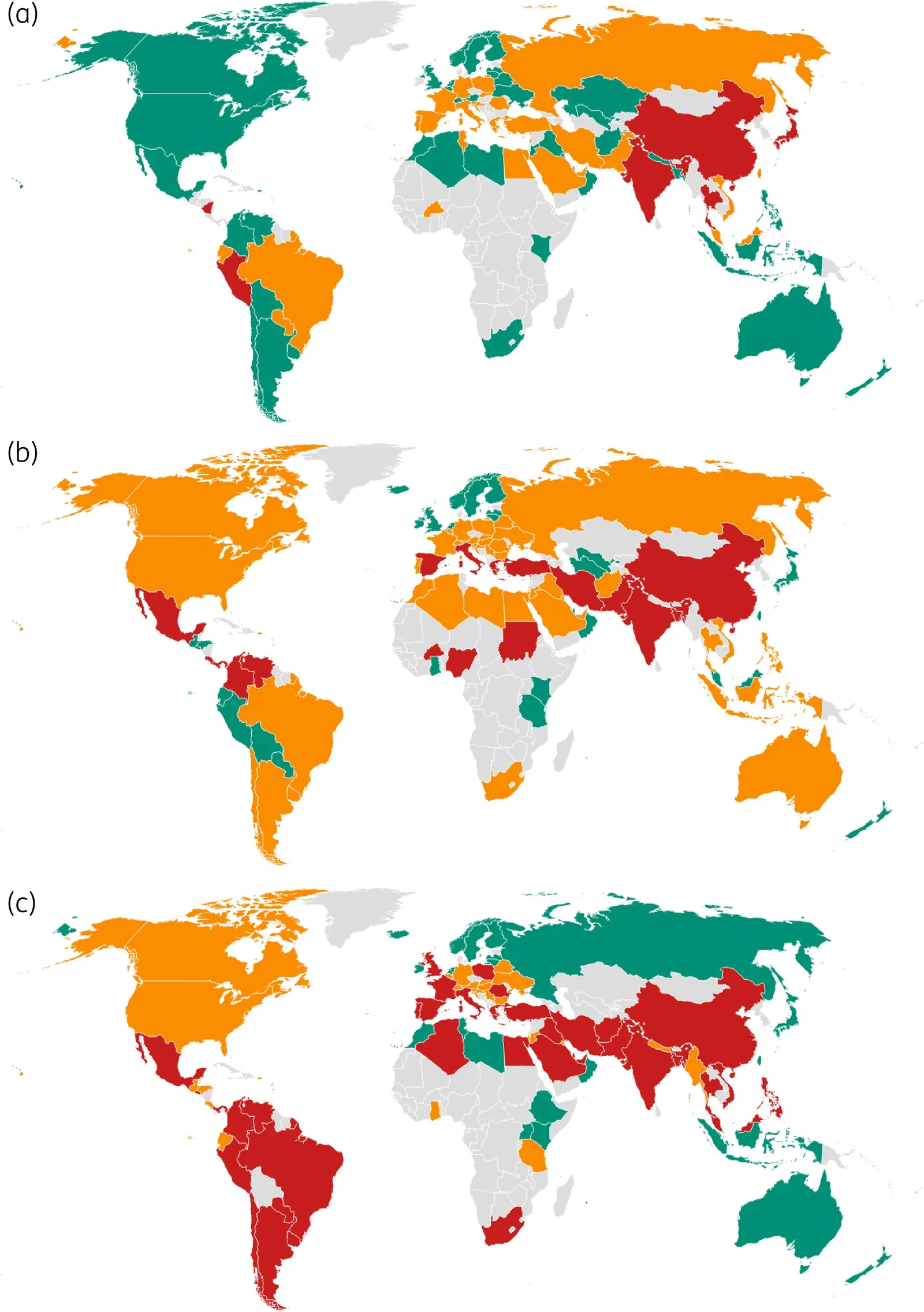
Global epidemiology of IMP (a), VIM (b) and NDM (c) MBLs across gram-negative species. Prevalence was estimated based on reports in published literature; additional methodological details on data sources and map construction are provided in the Supplementary material. Countries in red indicate widespread dissemination; countries in orange indicate potential for widespread dissemination; and countries in green indicate isolated reports or early emergence of the enzyme(s). Countries shown in grey represent regions where no reliable data are available. Figure created with Datawrapper (https://app.datawrapper.de).

VIM enzymes show a distinct global distribution, with high endemic-level prevalence in Italy, Greece, Spain, Turkey, Iran, Pakistan, Colombia, Venezuela, Brazil and Mexico (Figure 1b). Moderate prevalence is observed in countries such as France, Germany, South Africa, Egypt and Australia, whereas low or emerging prevalence is reported in Canada, the USA, Japan, Malaysia and much of Sub-Saharan Africa and Central America.^27,47,49–54^ Consistent with this geographic pattern, new VIM variants have been reported across geographically diverse locations, including Italy (VIM-1), France (VIM-2), Taiwan (VIM-3), Greece (VIM-4), Turkey (VIM-5), Singapore (VIM-6), the USA (VIM-7), Colombia (VIM-8) and both the UK and India (VIM-10).^27^ Among these, VIM-2 has demonstrated the broadest international dissemination, predominantly within high-risk clones of *P. aeruginosa* (e.g. ST235, ST111), which possess an enhanced capacity for acquiring exogenous resistance determinants. In Latin America, the *bla*_VIM_ gene has been detected in 47.6% of carbapenemase-producing *P. aeruginosa* isolates over a 5 year period.^52^ This is notable because in other regions, like the USA, VIM-producing *P. aeruginosa* remain rare (<2% of carbapenem-resistant *P. aeruginosa*).^53^ In contrast to VIM-2, VIM-1-like enzymes are more frequently encountered in Enterobacterales, with *K. pneumoniae* serving as the principal reservoir.

The global proliferation of NDM is notable (Figure 1c). In Southeast Asia and the Middle East, NDM are considered endemic. The prevalence of NDM in parts of Europe, South America, Central America and Canada is increasing across various gram-negative pathogens.^40,47,49–52,55^ In 2023, the European Centre for Disease Prevention and Control noted an exponential increase in NDM-harbouring *E. coli* occurring worldwide and specifically, a 6-fold increase in NDM-5 between 2013 and 2019 in the European Union.^56^ This spread is associated with high-risk clones such as ST137, ST405, ST410 and ST648, co-carriage with CMY- and CTX-β-lactamases.

In the USA, NDM prevalence has surged in specific geographic regions in recent years. Between 2016 and 2017, only 3% of carbapenemase-producing CRE in the USA were NDM.^57^ This increased to 9% between 2017 and 2019.^58^ Rates are pathogen specific. Among *Klebsiella, Enterobacter* and *Citrobacter* species, approximately 90% were *Klebsiella pneumoniae* carbapenamase (KPC)-producing compared to 53% for *E. coli.* In carbapenemase-producing *E. coli*, 35% harboured NDM enzymes. Among 30 390 carbapenem-resistant *P. aeruginosa* isolates, only 1.4% (422) were MBL-producing (414 VIM, 8 IMP).

Overall, CRE remain rare in the USA, as evidenced by the INFORM database data (2019–2021) where only 0.94% of 27 834 Enterobacterales isolates were carbapenem-resistant. In New York City, CRE rates increased by 14% from 2019 to 2024; NDM-producing isolates increased from *N* = 58 to *N* = 388 whereas KPC-producing isolates remained unchanged.^59^ Data collected from 2019 to 2023 across the USA corroborated these findings. In an age-adjusted incidence model, age-adjusted rates of NDM-producing CRE increased by 461% {incidence rate ratio [IRR], 5.61 [confidence interval (CI), 4.96–6.36]}.^60^ Most recently, the US Centers for Disease Control (CDC) reported on 1288 carbapenemase-producing CRE collected from 2016 to 2023 and found an increase in NDM-producing isolates from 5.4% in 2016 to 39.8% in 2023.^61^

Interestingly, combat zones and displacement of war-wounded refugees facilitate the rapid spread of MBL-producing infections, even to countries with historically low rates of antimicrobial resistance, such as the Netherlands.^62^ Indeed, a report of 5022 healthcare-associated infections from a total of 17 286 bacterial strains in Ukraine between June 2022 and December 2024 found 45.7% harboured an MBL (34.7% NDM-1, 29.6% VIM-type and 18.5% IMP-type).^63^ The most recent evidence underscores that the epidemiology of MBLs is dynamic, and global dissemination of multiple enzymes has been documented.

## Risk factors for acquisition and infection, diagnostics and clinical outcomes of MBL-producing gram-negative infections

Risk factors for acquisition of MBL-producing organisms mirror those described for other multidrug-resistant organisms, including intensive care unit (ICU) admission, mechanical ventilation, prior exposure to carbapenems, presence of central venous or urinary catheter and residence in nursing homes or long-term care facilities.^64,65^ In a prospective multicentre study of patients with gram-negative bloodstream infections across 19 Italian hospitals (June 2018 to January 2020), individuals with MBL-producing infections were older, often had comorbid conditions, including diabetes, cardiovascular disease, cancer or a history of transplantation, and more than half had been hospitalized within the preceding 90 days.^66^ Notably, 22.1% of cases were community-acquired, suggesting an underrecognized reservoir of MBL transmission at the time of hospital admission. For these reasons, clinicians should remain vigilant in testing carbapenem-resistant isolates for the presence of MBL enzymes as rapid epidemiologic shifts are commonly reported, even among patients without identifiable risk factors. A recent CDC report identified 21 cases of NDM-producing CRE from urinary sources in patients without healthcare exposure between September 2021 and September 2022.^67^ Of these patients, only 30% reported international travel within the prior year, and 88% were not hospitalized within 7 days of index culture collection.

Given the significant differences in substrate profile for MBLs as compared to serine carbapenemases, identification of the specific type of carbapenemase is essential to guide treatment decisions. The EDTA-modified carbapenem inactivation method utilizes EDTA as a chelating agent to distinguish MBL-producing Enterobacterales, but the test cannot identify which specific enzyme is present.^68^ Accordingly, MBL differentiation requires molecular detection, typically with multiplex polymerase chain reaction (PCR) assays or rapid lateral flow immunoassays that detect the most common carbapenemases, including NDM, IMP, VIM, KPC and OXA-48-like.^69^ Whole-genome sequencing is the gold standard for MBL identification and subtyping that would not be detected by PCR or immunoassays; however, this is unlikely to be available for most clinical laboratories, and results are typically not available early in clinical decision-making.

Clinical outcomes are generally poor for patients with MBL-producing infections, perhaps due in part to delayed time to active therapy secondary to limited carbapenemase detection methods, patient comorbidities and previous antibiotic exposures and limited antibiotic options. Kanj *et al.*^46^ reported prolonged hospital length of stay (LOS) among patients with NDM-producing infections (mean ± standard deviation: 22.24 ± 22.79 to 44 ± 28.2 days), significantly longer than patients without NDM-producing infections. ICU LOS is also longer for patients with NDM-1 compared to patients with infections caused by otherwise susceptible bacteria. Falcone *et al.*^66^ reported only 14.3% of patients with MBL-producing bloodstream infections received early appropriate antibiotic therapy and the attributable 30 day mortality was 35%, when compared with 5% for KPC-producing infections. Compared with bacteraemia due to carbapenem-susceptible bacteria, MBL-producing bacteraemia was independently associated with a higher 30 day mortality rate (adjusted odds ratio 5.86, 95% CI 2.72–12.76). This is consistent with other studies that report 30 day mortality rates of nearly 40%.^46^ Importantly, these associations hold true across other carbapenemase-producing pathogens. Among patients with carbapenemase-producing *P. aeruginosa* infections, the 30 day mortality rate was higher than those with non-carbapenemase-producing *P. aeruginosa* infections in both unadjusted (22% versus 12%; difference 9%, 95% CI 3–16) and adjusted analyses (difference 7%, 95% CI 1–14).^53^

## Treatment options

Historic treatment of MBL-producing infections parallels a paradigm established for treatment of KPC-producing infections prior to the development of highly active β-lactam/β-lactamase inhibitors. Specifically, combination regimens including salvage agents like colistin, tigecycline, fluoroquinolones and fosfomycin have been commonly employed, typically in the setting of preserved *in vitro* activity to these agents. Unlike KPC, however, aminoglycosides are not commonly used to treat infections caused by MBL-producing pathogens due to high rates of co-carriage of 16S rRNA methyltransferase genes conferring aminoglycoside resistance.^70^ The most important therapeutic option for MBL-producing infections is ATM given its unique stability against hydrolysis by MBLs.

The monobactam scaffold of ATM prevents productive catalytic positioning in the MBL active site through multiple mechanisms: (i) binding to the active site in an ‘inhibitor’ conformer which blocks or displaces the bridging water molecule required for hydrolysis, thereby preventing hydrolysis (‘nonproductive binding’), (ii) lack of bicyclic, or second, fused ring substituents found in penicillins, cephalosporins and carbapenems thwarting a productive recognition of the compound, and, (iii) presence of an N-sulphonate group on the β-lactam nitrogen which creates steric and electrical incompatibility for productive catalysis—though this latter mechanism is debated.^71–74^ ATM, however, remains vulnerable to hydrolysis by other β-lactamases, including extended-spectrum beta lactamases (ESBLs), AmpCs and serine-based carbapenemases, as these enzymes only require the β-lactam ring and associated carboxylate to form covalent bonds, both of which ATM possesses. ATM is also a substrate for antibiotic efflux pumps across gram-negative pathogens, particularly *P. aeruginosa*. Thus, many MBL-harbouring gram-negative pathogens demonstrate resistance to ATM, despite the inability of MBLs to hydrolyse ATM.

At least 11 regional or international guidelines provide treatment recommendations for serious, systemic infections caused by MBL-producing organisms.^75^ These documents generally position combination therapy with ceftazidime-avibactam plus ATM (CZA + ATM), ATM-avibactam (ATM-AVI), cefiderocol, fosfomycin, tetracyclines or polymyxins as potential therapeutic options. The 2026 Infectious Diseases Society of America Guidance suggests ATM-AVI or cefiderocol monotherapy as preferred treatment for NDM-producing Enterobacterales infections and cefiderocol for MBL-producing *P. aeurginosa*.^76^ By contrast, the 2022 European Society of Clinical Microbiology and Infectious Diseases (ESCMID) guidelines recommend cefiderocol for infections outside the urinary tract and suggest consideration of aminoglycosides for complicated urinary tract infections.^77^

### Ceftazidime-avibactam + aztreonam (CZA + ATM)

Avibactam is a non-β-lactam, β-lactamase inhibitor with a diazabicyclooctane (DBO) core that inhibits Ambler Class A, C and some Class D enzymes, but not Class B MBLs. When combined, AVI protects ATM from hydrolysis by co-produced serine β-lactamases, while ATM is stable to MBL-mediated degradation. This complementary mechanism allows ATM to reach its primary target, penicillin-binding protein 3 (PBP3), and exerts bactericidal activity.

Marshall *et al*.^78^ explored *in vitro* and *in vivo* models of the combination of CZA + ATM against 20 NDM-1-producing Enterobacterales. Using disk diffusion testing, 16 of 20 isolates demonstrated a zone of inhibition ≥21 mm to CZA + ATM, whereas only 1 isolate was susceptible to ATM alone and none were susceptible to CZA alone. In a murine neutropenic thigh infection model of NDM-1-producing *K. pneumoniae*, CZA + ATM resulted in an almost 4-log_10_-cfu/mL reduction at 24 hours compared to CZA alone. Biagi *et al.*^79,80^ reported synergy with ATM plus meropenem-vaborbactam (MVB) or imipenem-relebactam (I-R), although these combinations have not been used often in clinical practice because (i) CZA was commercially available prior to MVB or I-R, and AVI is a more potent inhibitor of non-KPC Class A and most Class C and D β-lactamases compared to vaborbactam and relebactam. In line, CZA displays higher rates of *in vitro* synergy with ATM as compared to MVB and I-R.

Global surveillance for susceptibility to CZA + ATM against MBL-producing pathogens is challenging as there is no standardized susceptibility testing method for the combination of these two drugs. In 2023, Harris *et al*.^81^ introduced a broth disk elution test, which was adopted by CLSI in 2025; this provides clinicians with a general idea of *in vitro* activity, but does not determine the minimum inhibitory concentration (MIC). This method looks for visible growth (not susceptible) or no growth (susceptible) at fixed concentrations of ATM 6 mg/L plus ceftazidime 6 mg/L plus AVI 4 mg/L. Susceptibility breakpoints for CZA + ATM have not been established by the Clinical & Laboratory Standards Institute (CLSI) or European Committee on Antimicrobial Susceptibility Testing (EUCAST) for Enterobacterales, *P. aeruginosa* or *S. maltophilia*. That said, CZA + ATM is expected to display relatively high rates of *in vitro* activity against MBL-producing Enterobacterales. Resistance to the combination has been documented amongst NDM-producing *E. coli* harbouring four-amino-acid insertions (YRIN/YRIK) in PBP3, which limit ATM binding. Treatment-emergent resistance to CZA + ATM has also been reported and is described in the ATM-AVI section below.

Much like Enterobacterales, CZA + ATM is likely active against *S. maltophilia,* displaying MICs 1–4-fold lower than ATM-AVI in a collection of 23 clinical isolates from the USA.^82^ However, isolate-level variation was observed by the extent of *in vitro* killing and bacterial regrowth, suggesting that CZA + ATM and ATM-AVI activity against *S. maltophilia* may vary between strains. In a collection of 27 MBL-producing *P. aeruginosa* isolates from Germany (19 VIM-2, 6 VIM-1, 3 IMP and 2 NDM), 19 (70%) of isolates had an MIC of ≥8 mg/L for CZA + ATM at a fixed concentration of 8 mg/L of ceftazidime; MICs were typically similar to ATM-AVI alone although for some VIM-producing isolates MICs of CZA + ATM were 1–3 dilutions lower.^83^ CZA + ATM is not *in vitro* active against *A. baumannii* since ATM has poor permeability in *Acinetobacter spp*., which along with the presence of intrinsic chromosomal AmpC enzymes negates any intrinsic activity.

The first report of clinical use of CZA + ATM was in 2015 for an NDM-9-producing *E. coli* bacteraemia in a patient with refractory acute lymphoblastic leukaemia.^84^ In 2016, the first report of a paediatric patient treated with CZA + ATM for recurrent *S. maltophilia* bacteraemia was published.^85^ In 2020, Falcone *et al.*^86^ performed a prospective, observational study of 102 adult patients admitted to 3 hospitals in Italy and Greece with bloodstream infections due to MBL-producing Enterobacterales (82 NDM and 20 VIM). Fifty-two patients (47 NDM and 5 VIM) received CZA + ATM (CZA 2.5 g every 8 hours and ATM 2 g every 8 hours), which was determined to be *in vitro* active by double-disk synergy testing and gradient-test methods. Fifty patients received other active antibiotics (OAA). The overall mortality rate at 30 days was significantly lower in CZA + ATM-treated patients compared to OAA (19.2% versus 44%; *P* = 0.007). In the OAA group, mortality in patients receiving colistin-based regimens was 59% (16/27) compared to 26% for non-colistin regimens (6/23). A multivariate Cox regression model showed treatment with CZA + ATM was independently associated with lower mortality after controlling for other clinically relevant factors [hazard ratio (HR), 0.17 (95% CI, 0.07–0.41); *P* < 0.001]. The combination was also associated with lower rates of clinical failure at day 14 [HR, 0.30 (95% CI: 0.14–0.65)] and shorter lengths of stay from time of positive blood culture [HR: 0.49 (95% CI: 0.30–0.82)].

In a subsequent report, Falcone *et al.*^87^ published a prospective, observational study of consecutive, adult patients hospitalized at a single centre in Italy with an MBL-producing Enterobacterales infection from January 2019 to October 2022. A total of 342 patients were included (328 NDM- and 15 VIM-producing strains). Most patients had bloodstream infections (*n* = 199), followed by pneumonia (*n* = 60) or urinary tract infections (*n* = 60). Among the NDM-producing isolates, 99.4% were *K. pneumoniae.* For patients who survived 48 hours, 62.7% received CZA + ATM (*n* = 215), 9.6% received cefiderocol-containing regimens (*n* = 33), 7.6% received colistin-containing regimens (*n* = 26) and 10.8% received OAA (*n* = 37). Adjunctive susceptibility testing showed that only 33.2% of all isolates were fully susceptible to cefiderocol by disk diffusion testing and that synergy for CZA + ATM was observed in 99.7% of isolates. The overall 30 day mortality was 29.7%, which was highest among patients with pneumonia (41.7%). Receipt of *in vitro* active antibiotics within 48 hours was protective against mortality [adjusted HR (aHR) 0.48 (95% CI 0.26–0.8); *P* = 0.007] overall; however, CZA + ATM was the only antibiotic regimen independently associated with a reduced 30 day mortality rate [aHR 0.39 (95% CI, 0.18–0.86); *P* = 0.02]. Thirty-day mortality in patients receiving colistin-containing regimens was 50% (13/26) compared to 33% (11/33) for cefiderocol, 22.3% (48/215) for CZA + ATM and 13.5% (3/37) for OAA. Of patients receiving CZA + ATM, 9 (4%) experienced a severe adverse event (2 pancytopenias, 2 allergic reactions, 1 seizure, 4 increased liver function tests); 35% of patients receiving colistin experienced acute kidney injury.

Data demonstrating the efficacy of CZA + ATM are specific to MBL-producing *K. pneumoniae*, and this combination should not be presumed to be equally effective against other MBL-producing pathogens, including *E. coli, P. aeruginosa* and *S. maltophilia.* Indeed, the clinical data for treatment of MBL-producing *P. aeruginosa* with CZA + ATM is limited to a single-centre case series of 8 patients with VIM-producing *P. aeruginosa.*^88^ Importantly, the MICs for ATM alone and CZA + ATM were the same across all isolates, suggesting no synergy for the combination against *P. aeruginosa.* Overall, 87% (7/8) of patients were successfully treated, but 25% (2/8) died within 28 days. Similar MICs between ATM alone and CZA + ATM were also observed in a study of 153 multidrug-resistant (MDR) *P. aeruginosa* isolates in Germany.^89^ In more dynamic time-kill and hollow-fibre infection model studies, synergistic killing of CZA + ATM for some MBL-producing *P. aeruginosa* at sub-inhibitory concentrations has been documented.^90,91^ Taken together, CZA + ATM may be a potential salvage regimen for MBL-producing *P. aeruginosa*, particularly VIM-producing isolates, but activity is less reliable than in Enterobacterales due to additional mechanisms of ATM resistance present (e.g. efflux, porin loss). Clinical experience for CZA + ATM for *S. maltophilia* remains limited to case reports.^92–97^

Optimized dosing and infusion strategies are not standardized across centres using the combination of CZA + ATM. Lodise *et al.*^98^ conducted a study using the hollow-fibre infection model to simulate clinically relevant exposures against NDM-producing *E. coli* and *K. pneumoniae* isolates. Concomitant administration of both agents provided enhanced early bacterial killing compared to staggered dosing; however, at 168 hours, killing was similar between dosing strategies for both *K. pneumoniae* and *E. coli*. The rate and extent of bacterial killing was greatest with continuous infusion compared to 2 hour infusions; 30 minute infusion times resulted in no bacterial killing. For ATM, regimens of 8 g/day via continuous infusion or as 2 hour infusions every 6 hours demonstrated improved bacterial killing and resistance suppression relative to 6 g/day regimens divided every 8 hours; however, the 8 g/day regimen has been associated with potential hepatotoxicity in a Phase 1, open-label, healthy volunteer study.^99^ The Phase III ASSEMBLE study demonstrated hepatotoxicity in patients receiving ATM at a daily dose of 6 g/daily.^100^ Thus, the recommended dosage for most clinical scenarios in patients with a creatinine clearance >50 mL/min is CZA 2.5 g IV every 8 hours over 3 hour infusion PLUS ATM 2 g IV every 8 hours over 3 hour infusion administered simultaneously (Table 2). Notably, we do not know how these drugs were administered in the available clinical data. Concomitant administration requires multidisciplinary coordination between prescribers, pharmacists and nursing to ensure operational consistency throughout the treatment course.

**Table 2.** Considerations for CZA + ATM or ATM-AVI

	CZA + ATM	ATM-AVI
Dose	2.5 g IV q8h CZA + 2 g IV q8h ATM	2.67 g loading dose once, followed by 2 g (1.5 g ATM, 0.5 g AVI) every 6 hours
Administration	Package insert states 30 minute infusion for ATM, 2 hours for CZA Clinical studies prefer 3 hour infusions; some studies suggest 8 hour infusions More complex preparation, administration and stewardship coordination Consider simultaneous infusion of both drugs to optimize early bacterial killing More operational burden (extra IV access, compatibility/logistics, pharmacy complexity)	Standard 3 hour infusion Less pharmacy and nursing time Less plastic waste Less fluid per day
Susceptibility testing	Provisional broth disk elution method	Gradient diffusion strip Disc diffusion (30/20 µg ATM/AVI) Broth microdilution
	In a collection of 37 MBL-producing Enterobacterales, ATM-AVI MICs were within one 2-fold doubling dilution of ATM-AVI plus ceftazidime, at a fixed concentration of 4 mg/L AVI, regardless of ceftazidime concentration.^101^ There was also high agreement between Liofilchem test strips for ATM-AVI with combination testing methods for CZA + ATM against Enterobacterales.^102^ ATM-AVI susceptibility testing may be a reasonable surrogate for CZA + ATM for Enterobacterales only.	
Adverse reactions	Higher doses of ATM are associated with greater likelihood of hepatic injury; both options utilize 6 g ATM/day. Definitions of hepatic impairment and hepatic injury are inconsistent across published studies. Side effect profiles are similar and include infusion reactions, rash, hypokalaemia, diarrhoea, nausea, anaemia and neutropenia The additive toxicity of including ceftazidime is unknown, and there are no direct comparative safety data between the two options.	
Extended infusion stability	Stable up to 12 hours at room temperature; up to 72 hours refrigerated. Not stable for 24 hours at body temperature^103^	Stable up to 12 hours at room temperature One case report of administering total daily dose continuously as two, 12 hour infusions for OPAT^104^
Cost	Varies by country and institutional contracts. Currently, generally cost neutral between the two options in the USA. Generic options and global market availability will drastically impact pricing.	
In vitro activity against MBL-producing pathogens	No clinically relevant differences have been described to date	
Other considerations	More published clinical data and real-world experience for MBL-producing Enterobacterales ‘Target redundancy’	More AVI exposure and PK/PD optimized Availability globally is more limited that CZA + ATM Supported by randomized clinical data, although limited published use in MBL-producing infections

### Aztreonam-avibactam

ATM-AVI began clinical development in 2012 and was approved by the European Medicines Agency in 2024 and the Food and Drug Administration (FDA) in 2025.^105^ The EUCAST, CLSI and FDA-approved susceptibility breakpoints are ≤4/4 mg/L for Enterobacterales; however, a susceptibility breakpoint of ≤8 mg/L was the provisional breakpoint used in clinical development. In a study of Enterobacterales collected between 2019 and 2023 from 63 countries, 93.1% (498/535) of MBL-producing *E. coli* and 99.8% (2171/2176) of MBL-producing *K. pneumoniae* demonstrated ATM-AVI MICs ≤8 mg/L.^106^ The proportion of *E. coli* inhibited dropped to 78.9% when an ATM-AVI MIC cut-off of ≤4 mg/L was applied.

In a large collection of 1400 clinical *S. maltophilia* isolates also from US medical centres, ATM-AVI inhibited 99.6% of isolates at an MIC ≤8 mg/L with an MIC_50/90_ of 2/4 mg/L.^107^ A hollow-fibre model utilizing human simulated conditions against 3 clinical *S. maltophilia* isolates determined that only continuous infusion strategies of ATM-AVI with higher ATM exposure at 8 grams/day achieved durable bactericidal activity; 6 g/day of ATM did not achieve sustained bactericidal activity when used in ATM-AVI or CZA + ATM combinations. The addition of ceftazidime did not improve killing activity, but was associated with greater β-lactamase induction via *bla*L1 upregulation.^108^ Conflicting data and isolate-level heterogeneity in *S. maltophilia* make positioning of ATM-AVI in treatment paradigms challenging for this pathogen. As mentioned in the CZA + ATM section, it is important to note that the addition of AVI to ATM does little to restore ATM’s activity against MBL-producing *P. aeruginosa* (MIC_90_ ATM-AVI 32 mg/L, MIC_90_ ATM 64 mg/L) due to the presence of non-enzyme-mediated resistance in this pathogen.^109^ Like CZA + ATM, ATM-AVI is not *in vitro* active against *A. baumannii*.

ATM-AVI is formulated as a fixed-dose product containing a 3:1 ratio of ATM to AVI. Avibactam exposures were optimized to partner with ATM (which is not the case with CZA-ATM). Labelled dosing in patients with normal renal function provides for a single loading dose of 2.67 g (2 g ATM, 0.67 g AVI) over 3 hour infusion followed by a maintenance dose of 2 g (1.5 g ATM, 0.5 g AVI) every 6 hours over 3 hour infusion (Table 2). This dosing strategy provides more AVI per day and improved pharmacokinetic performance relative to the Infectious Diseases Society of America (IDSA)-recommended CZA + ATM dosing scheme described earlier (predicated joint probability of target attainment of 89 to >99% across all renal function groups for ATM-AVI compared to 84% for CZA + ATM at an MIC of 8 mg/L for Enterobacterales).^110,111^

Notably, the regulatory-approved loading dose is different than the original 500/167 mg, 30 minute infusion loading dose followed immediately by a 1500 mg/500 mg, 3 hour infusion dose studied in the Phase 2a, open-label REJUVINATE study.^112^ During conduct of the Phase 3 studies, it was recognized that this loading dose had the potential for dosing administration errors in clinical practice, so a simplified loading dose was evaluated using pharmacokinetic/pharmacodynamic (PK/PD) simulations and was eventually approved.

The primary clinical data to support approval of ATM-AVI are derived from the REVISIT study, a phase 3, randomized, global trial comparing ATM-AVI (±metronidazole) versus meropenem (±colistin) in patients with complicated intra-abdominal infection or pneumonia.^113^. The primary conclusion from the study was that ATM-AVI was non-inferior to meropenem. Unfortunately, only 80 isolates from patients in the study were tested for carbapenemases, and of these, only 12.5%^10^ were MBL producers (including one *S. maltophilia).* Clinical response was 28.6% (2/7) with ATM-AVI and 66.7% (2/3) with meropenem. Based on CLSI breakpoints, clinical cure was achieved in 66.7% (20/30) versus 64% (16/25) for ATM-AVI versus meropenem in patients with ATM-non-susceptible isolates, and 46.2% (6/13) versus 37.5% (3/8) in patients with meropenem (MEM)-non-susceptible isolates, respectively.

The ASSEMBLE trial by Daikos *et al*.^100^ was a prospective, multicentre, open-label trial where hospitalized adult patients were randomized 2:1 to ATM-AVI or best available therapy (BAT) for 5–14 days for confirmed MBL-producing pathogens. Only 15 patients were enrolled in the study, of whom 3 received BAT, so the data should also be interpreted with caution due to sample size. Clinical cure rates at test of cure were 5/12 (42%) for ATM-AVI and 0/3 (0%) for BAT. Twenty-eight-day all-cause mortality rates were 1/12 (8%) for ATM-AVI and 1/3 (33%) for BAT. Favourable microbiologic responses in the ATM-AVI group were 6/12 for Enterobacterales, 0/2 for *P. aeruginosa* and 1/3 for *S. maltophilia.* Clinical cure was not achieved in any of the 3 *E. coli* isolates in the ATM-AVI group.

Due to the lack of observational studies and very limited number of published case reports for ATM-AVI, the real-world clinical data for CZA + ATM previously described offer the strongest support for the efficacy of ATM-AVI. Laffont-Lozes *et al*.^114^ reported switching a patient from CZA + ATM to ATM-AVI administered via continuous infusion for an OXA-48 and NDM-1 producing *K. pneumoniae* meningitis and brain abscess. Keck *et al.*^115^ treated a 55 year-old female with ATM-AVI (eventually stepped down to eravacycline for transitions-of-care) for a complex orthopaedic infection with hardware due to NDM-producing *E. cloacae* with clinical success at 8 weeks. Mazzitelli *et al.*^116^ treated four cases of bacteraemia with NDM-1-producing, cefiderocol-resistant *K. pneumoniae* with ATM-AVI, all with clinical success.

Reduced susceptibility to ATM-AVI is conferred by similar mechanisms as resistance to CZA + ATM, especially in NDM-5-harbouring *E. coli* (which are more likely to also harbour PBP3 insertions), and particularly among ST167, ST410, ST405 and ST361 clones.^117^ Elevated MICs are mediated by PBP3 amino acid insertions (YRIN or YRIK) and co-production of extended-spectrum cephalosporinases, including CTX-M-199, CMY-141, CMY-145 and CMY-42-like enzymes.^107,118,119^ PBP3 is the primary binding target for ceftazidime, cefiderocol and ATM, and thus isolates possessing PBP3 mutations often exhibit elevated ATM-AVI and cefiderocol MICs. In *E. coli*, resistance to ATM-AVI can be multifactorial with co-production of CMY-type enzymes and PBP3 insertions driving increased MIC values.^120^ CMY-type enzyme co-production is most common amongst NDM-5-harbouring *E. coli*, but can co-produced with other NDM subtypes and other carbapenemases and ESBLs as well.^119^ Of note, in a collection of 65 *E. coli* harbouring YRIN or YRIK insertions, 16 (25%) had an MIC ≤4 mg/L, which would be considered susceptible per all current breakpoints. In a collection of 40 NDM-producing *E. coli* isolates from the USA, 30 (75%) harboured a PBP3 mutation; of these, only 12 (40%) were resistant (MIC ≥8 mg/L).^121^ Castanheira *et al*.^119^ describe a collection of 62 global *E. coli* isolates harbouring PBP3 insertions where ATM-AVI inhibited 80.6% of isolates at ≤4/4 mg/L; isolates that co-harboured CMY cephalosporinases displayed higher MICs. The clinical impact of treating an isolate that harbours a PBP3 insertion but tests *in vitro* susceptible to ATM-AVI is unknown.

In *Enterobacter* spp., mutations in AmpC regulatory genes, ampC attenuator region and AmpC structural variations resulted in high-level ATM-AVI resistance.^122^ Treatment-emergent resistance to ATM-AVI has also been described in a patient with NDM-1-producing *Providencia* spp.^123^ Resistance was attributed to a single amino acid insertion (glycine at residue 420) in PBP3 that is distinct from the aforementioned 4-amino acid PBP3 insertions found in *E. coli.* Mutations in the porin gene *ompC* were also identified that may have an impact on the permeability of ATM-AVI across the cell membrane. Importantly, ATM-AVI resistance has been identified most in NDM-producing *E. coli* across diverse clonal lineages, suggesting that *E. coli* can tolerate PBP3 insertions without a fitness trade-off.^120,121,124^ By contrast, PBP3 insertions in *K. pneumoniae* are associated with a marked fitness cost, which may explain the relative absence of such mutations in clinical practice.^125^ Only recently have PBP3 insertions been reported in KPC-producing *K. pneumoniae* from a single study in Taiwan.^126^

Mutations in PBP2 have also been identified in *E. coli;* however, cephalosporins and ATM weakly bind to PBP2, and thus, the clinical significance of these mutations to ATM-based combinations is unclear.^127^ In vivo resistance development in an OXA-244-harbouring *E. coli* was linked to PBP3 insertions plus mutations in *acrB* and PBP2.^128^ PBP2 mutations in NDM-producing *E. coli* may impact activity of novel inhibitors such as durlobactam and zidebactam.^129,130^

Simner *et al*.^131^ described a patient who received a kidney transplant in India with subsequent pyelonephritis due to NDM-5-producing ST167 *E. coli*. The isolate was resistant to CZA + ATM without any previous drug exposure and harboured *bla*_CMY-59_ encoding an expanded-spectrum cephalosporinase, mutations in the *cirA* iron transport gene contributing to cefiderocol resistance and a 4-amino acid duplication in PBP3 (YRIN) preventing ATM binding. Haidar *et al*.^132^ described treatment-emergent resistance to CZA + ATM in an ST361 *E. coli* harbouring NDM-5, *bla*_CMY-145_ and PBP3 (encoded by *ftsl*) mutations with elevated ATM-AVI MICs at baseline (16/4 mg/L), but after exposure to CZA + ATM, new mutations in *ftsl* and *bla*_CMY-145_ emerged resulting in an MIC of >128/4 mg/L. This underscores the importance of antimicrobial susceptibility testing and surveillance for all isolates, particularly since whole-genome sequencing and NDM-typing are not readily available to most clinicians globally.

### Is CZA + ATM or ATM-AVI preferred for treatment of MBL-producing Enterobacterales?

Practically speaking, most centres will need to adopt a unified approach to MBL treatment, leveraging either the combination of CZA + ATM or ATM-AVI as the preferred option. There are pros and cons with each approach, principally centred around the potential utility of ceftazidime as part of the combination. It has been proposed that ceftazidime offers additional benefit (e.g. ‘target redundancy’) by binding to alternative PBPs (ATM has high affinity for PBP3 and ceftazidime can bind PBP1a/b at concentrations ≥4 mg/L).^133,134^ While perhaps somewhat counterintuitive, it is possible that some fraction of ceftazidime remains free to acylate PBPs despite being readily hydrolysed by NDM enzymes, and therefore the combination may provide additive activity in situations when ATM is rendered ineffective. Whether this translates into clinical utility is unknown.^134,135^ Notably, the presence of ceftazidime may also induce other enzyme production as previously shown in *S. maltophilia*.^108^

The primary disadvantage of CZA + ATM is the co-administration barriers that may increase the probability of medication errors, suboptimal exposures and additive pharmacist and nursing time. The coformulation of ATM-AVI circumvents these challenges and results in less plastic waste than CZA + ATM.^136^

### Cefiderocol

Cefiderocol is a siderophore cephalosporin with a distinctive chemical architecture that combines structural elements of ceftazidime and cefepime.^137,138^ At the C-7 position, a carboxypropyl-oxyimino group and aminothiazole ring confer potent antipseudomonal activity and facilitate penetration of the gram-negative outer membrane. At C-3, a zwitterionic methylpyrrolidinium moiety, analogous to that of cefepime, reduces affinity for β-lactamase active sites. In addition, a chlorocatechol substituent at C-3 functions as a siderophore, chelating extracellular iron and promoting active transport via bacterial iron uptake systems, thereby increasing periplasmic drug concentrations using a ‘Trojan horse’ mechanism. Cefiderocol inhibits cell wall synthesis through binding primarily to PBP3.^139^

Surveillance data demonstrate variable *in vitro* activity of cefiderocol against MBL-producing Enterobacterales. Notably, cefiderocol susceptibility is highly influenced by testing method and is not often tested in clinical laboratories.^140^ The susceptibility breakpoint for Enterobacterales and *P. aeruginosa* varies between CLSI and FDA (≤4 mg/L) compared to EUCAST (≤2 mg/L). In the SENTRY programme (2020–2021), carbapenem resistance was identified in 2.8% (442/16 065) of Enterobacterales isolates, with a cefiderocol MIC_90_ of 4 mg/L among CRE.^141^ Against 52 MBL-producing isolates, 96.6% were susceptible according to CLSI and FDA breakpoints; however, it is now recognized that *in vitro* activity is dependent on the type of MBL identified and host organism. In a study of 204 NDM from China, the MIC_50/90_ for 98 *K. pneumoniae* was 4/16 mg/L, for 44 *E. coli* it was 4/32 mg/L and for 45 *Enterobacter* spp. it was 4/>64 mg/L. Most resistant *E. coli* harboured PBP3 mutations, whereas resistance in *K. pneumoniae* was due to *cirA, fepA* and *fhuA* alterations.^142^ In a Public Health England reference collection, cefiderocol inhibited 95.7% of VIM-producing Enterobacterales, but only 72.1% of NDM producers at the CLSI breakpoint.^143^

For *P. aeruginosa,* cefiderocol inhibited 80% of IMP-producing and 72.7% of NDM-producing *P. aeruginosa* at the CLSI breakpoint.^143^ In the ATLAS surveillance programme, cefiderocol was active against 93.9% (835/890) of carbapenem-resistant *P. aeruginosa.* Amongst the 133 MBL-producing *P. aeruginosa* isolates, cefiderocol had an MIC ≤4 mg/L for 100% of VIM (*n* = 68) and IMP (*n* = 21) positive isolates, but only 72.3% of NDM (*n* = 47) positive isolates.^144^ Notably, NDM-1 and NDM-5 are able to hydrolyse cefiderocol.^137^ While there appears to be an *in vitro* advantage of ATM-AVI over cefiderocol for Enterobacterales, cefiderocol is more likely to display activity against MBL-producing *P. aeruginosa* due to increased likelihood of non-β-lactamase mechanisms of ATM resistance.^145^ Both drugs display decreased susceptibility to NDM-harbouring isolates due to the emergence of PBP mutations.

Cefiderocol also displays *in vitro* activity against MBL-producing *S. maltophilia* and *A. baumannii*. In the ATLAS programme, cefiderocol inhibited 88.5% of carbapenem-resistant *A. baumannii* at the CLSI breakpoint of ≤4 mg/L; however, they did not describe how many of these isolates harboured MBLs.^144^ In the SIDEO-WT surveillance study which collected approximately 46 000 gram-negative clinical isolates from North America and Europe over 5 years, only 25 MBL-producing *A. baumannii* were identified with cefiderocol susceptibility rates of 60% and 44% by CLSI and EUCAST breakpoints (≤2 mg/L), respectively.^146^Among a collection of 2030 *S. maltophilia* isolates, cefiderocol inhibited 98.6% at an MIC ≤1 mg/L (the current CLSI breakpoint).^147^

Clinical efficacy data for cefiderocol for the treatment of gram-negative infections have been derived from three randomized trials. CREDIBLE-CR, a phase 3, open-label study, randomized 152 patients with carbapenem-resistant infections to cefiderocol or BAT.^148^ Fourteen-day all-cause mortality was 19% (19/101) in the cefiderocol arm versus 12% (6/49) with BAT. APEKS-NP compared cefiderocol with high-dose meropenem for nosocomial pneumonia and demonstrated non-inferiority between treatment arms, with 14 day mortality of 12.4% versus 11.6%, respectively.^149^ Pooled analysis identified a total of 34 patients with MBL-producing pathogens across both studies.^150^ In CREDIBLE-CR, clinical cure at test of cure was numerically higher with cefiderocol than BAT [75.0% (12/16) versus 28.6% (2/7)], whereas cure rates in APEKS-NP were similar between treatments [62.5% (5/8) versus 66.7% (2/3)]. Among cefiderocol-treated patients, cure rates were numerically lower for NDM-producing infections (56.3%, 9/16) compared with non-NDM infections (100%, 8/8). Twenty-eight-day mortality across both trials was 12.5% (3/24) with cefiderocol and 50.0% (5/10) with BAT.

The GAME CHANGER trial evaluated cefiderocol versus standard care for gram-negative bloodstream infections.^151^ Overall 14 day mortality met the pre-specified non-inferiority criteria (8% versus 7%); however, among patients with MBL-producing infections, mortality was higher with cefiderocol at both 14 days [31% (5/16) versus 0%] and 30 days [50% (8/16) versus 18% (2/11)]. As cefiderocol susceptibility rates were broadly comparable across studies, the reasons underlying divergent clinical outcomes remain uncertain, particularly as specific MBL genes were not reported.^152^ Other studies report clinical cure with cefiderocol treatment despite patients having cefiderocol-resistant isolates.^153^

In addition to case reports, a real-world observational cohort study of 239 patients with gram-negative bacterial infections in Italian hospitals treated with cefiderocol has been reported.^154,155^ The cumulative probability of 30 day survival was 71%, with no differences observed amongst patients receiving cefiderocol monotherapy or in combination with other antibiotics. NDM-producing *Klebsiella* spp. infection was associated with higher mortality (aHR: 6.22, 95% CI: 2.09–18.50, *P* = 0.001).

Resistance to cefiderocol was initially attributed to mutations in iron transporter genes like *piuA, piuD* and *pirA* in *P. aeruginosa*; c*irA, fiu* and *piuA* in Enterobacterales; and *piuA* and *pirA* in *A. baumannii*. Resistance has also been attributable to β-lactamase hydrolysis. Warecki *et al.*^137^ showed that NDM-1 and NDM-5 efficiently hydrolyse cefiderocol, whereas VIM-2 and IMP-1 display markedly reduced catalytic efficiency. In this context, NDM-5 hydrolyses cefiderocol and ceftazidime with equal efficiency. NDM-1, NDM-5, VIM-2 and IMP-1 form a reversible enzyme-product adduct with cefiderocol, but the stability of this adduct differs across MBL enzymes. In IMP-1 and VIM-2, loop L3 adopts a closed conformation over the active site, creating steric hindrance that slows product release and results in effective inhibition. In contrast, NDM variants maintain L3 in a more open configuration, allowing rapid dissociation of the product and high turnover rates, which explains their retained ability to hydrolyse cefiderocol. Consistent with this mechanism, surveillance data suggests that MICs for cefiderocol are elevated in NDM-producing bacteria relative to other MBLs.^137,156,157^ Accordingly, cefiderocol resistance may reflect a variety of factors, including mutations in the CirA siderophore transporter that reduce drug uptake, NDM enzymes that efficiently hydrolyse cefiderocol and porin deficiencies (e.g. *ompK35*/*ompK36*) that slow drug transport across the cell membrane, particularly for *K. pneumoniae.*^158–160^ In addition, PBP3 insertions (seen exclusively in *E. coli*) may result in cross-resistance between cefiderocol, CZA + ATM and ATM-AVI.^131^ Cefiderocol resistance has also been observed in patients with NDM-1-producing *K. pneumoniae* that did not harbour PBP3 mutations. Addition of xeruborbactam restored cefiderocol susceptibility suggesting that NDM-1 hydrolyses cefiderocol.^116^

### Non-β-lactam antibiotics

Colistin, polymyxin B, fosfomycin, tigecycline, eravacycline, minocycline, ciprofloxacin, levofloxacin, trimethoprim-sulfamethoxazole and nitrofurantoin (cystitis only) may be considered for treatment of MBL-producing gram-negative infections, often as combination therapy, if *in vitro* susceptible and the agent is appropriate for the pathogen and infection type. Collectively, these agents encompass ‘BAT’ and ‘other active antibiotic’ comparators used prior to the availability of novel β-lactam-based therapies for MBL-producing infections. Unfortunately, MBL-producing pathogens exhibit high rates of resistance to these agents limiting their overall impact on clinical practice.

In the USA, the most common source of NDM-producing pathogens is urine cultures (sometimes in patients without healthcare exposure) or other surveillance cultures. In fact, only a minority of cases are associated with systemic or invasive disease.^57,67,161^ While the latter dominates dialogue and recommendations around NDM treatment, NDM-producing cystitis can successfully be treated with nitrofurantoin or ciprofloxacin if *in vitro* susceptibility is displayed. In cases of complex intra-abdominal infection without bacteraemia and source control, tigecycline or eravacycline monotherapy may be considered on a case-by-case basis.^162^ The 2026 IDSA guidance supports the use of tigecycline or eravacycline as alternative options for NDM-producing Enterobacterales not in the bloodstream or urinary tract.^76^

Intravenous fosfomycin has been used globally for years and recently gained regulatory approval in the USA following the results of the ZEUS trial.^163^ No patients with MBL-producing infections were included in the trial. As such, most evidence in support of IV fosfomycin stems from observational studies where fosfomycin is often used in combination. The ESCMID guidelines for multidrug-resistant gram-negative infections state there is no evidence to recommend for or against fosfomycin, but that it should be used in combination with more than one *in vitro* active drug if newer β-lactams are either not susceptible or available.^77^ The guidelines make no recommendation for or against specific combinations. The 2026 IDSA guidance document recommends fosfomycin for CRE urinary tracy infections (UTIs), but does not specifically comment on the use of fosfomycin for MBL-producing infections.^76^ It is the opinion of the authors that fosfomycin is not optimal for treatment of MBL-producing *P. aeruginosa,* although it may be a reasonable adjunct therapy in combination with other agents based on the potential for synergistic activity.^164,165^

As with β-lactam antibiotics, the lack of available, reliable and practical antibiotic susceptibility testing methods may impact clinical decision-making. Susceptibility testing for fosfomycin or colistin/polymyxin is particularly challenging and rarely available for clinical laboratories.

## β-Lactam enhancer combinations

### Cefepime-zidebactam

Cefepime-zidebactam (FEP-ZID) received FDA approval in 2026 for treatment of complicated urinary tract infections. Zidebactam is a novel bicyclo-acyl hydrazide derivative of the DBO β-lactamase inhibitors that inhibit Ambler class A and C serine β-lactamases. Despite zidebactam’s inability to inhibit MBL enzymes directly, which would be expected to readily hydrolyse cefepime, synergistic killing has been observed with FEP-ZID across various gram-negative pathogens due to an enhancer effect.^166,167^ PBP2 is ubiquitous across gram-negative pathogens, highly conserved with infrequent mutations due to high organism fitness cost, and it plays a central role in cell shape maintenance and lysis.^168,169^ With zidebactam, the DBO scaffold was modified to augment binding affinity for PBP2, which serves as a complementary killing target to cefepime’s PBP3. One suggested hypothesis for the ‘enhancer’ phenomenon is that cefepime’s rate of binding and ability to acylate PBP3 exceeds its rate of MBL-mediated hydrolysis; however, to date this has only been demonstrated in VIM-1-producing *P. aeruginosa*.^170^ Other enhancer agents are in development, each of which demonstrates intrinsic activity against *Enterobacterales* and other gram-negative pathogens through PBP2 inhibition, but with varying potencies.^171–173^

Zidebactam alone inhibits MBL-producing Enterobacterales with varying potency. The MIC_90_ of zidebactam alone against 50 *E. coli* isolates was 0.5 mg/L, whereas the MIC_90_ against 58 *Klebsiella* spp. isolates was 32 mg/L.^174^ It is not active against Proteeae (i.e. *Morganella morganii, Providencia* spp., *Proteus* spp.) as demonstrated by MICs ≥32 mg/L for all isolates tested.^174^

Given the unique characteristics of zidebactam as a PBP2 enhancer, the combination of FEP-ZID is tested as a fixed ratio rather than as a fixed concentration of β-lactamase inhibitor. CLSI and EUCAST breakpoints have not been established for FEP-ZID. A provisional susceptibility breakpoint of ≤64 mg/L was set by CLSI based on pre-clinical PK-PD studies; however, further assessment is ongoing.^175^ The FDA-approved breakpoint is ≤8 mg/L for Enterobacterales and *P. aeruginosa* at a dose of 3 g (2 g cefepime + 1 g zidebactam) IV every 8 hours, over 1 hour infusion, based on the labelled indication of complicated urinary tract infection.

Amongst 1018 carbapenem non-susceptible *Enterobacterales,* neither cefepime nor zidebactam demonstrated significant *in vitro* activity alone (MIC_90_ >64 mg/L for both); however, the combination of FEP-ZID showed a 32-fold decrease in MICs and an overall MIC_90_ of 4 mg/L, which was the same MIC_90_ for the subset of MBL-producing isolates (*n* = 191).^176^ Among 593 MBL-producing *Enterobacterales* isolates in France, the MIC_90_ for FEP-ZID was 1 mg/L.^117^ Against 28 NDM-producing *E. coli* with PBP3 insertions that all co-produced CMY-42, the MIC_90_ of FEP-ZID was 0.25 mg/L, compared to MIC_90_ = 1 mg/L for zidebactam alone.^177^ In a separate collection of 39 NDM-producing *E. coli* isolates all harbouring PBP3 four-amino-acid insertions from India, FEP-ZID inhibited 100% of isolates at an MIC ≤1 mg/L.^178^

Among 94 MBL-positive *P. aeruginosa,* FEP-ZID had an MIC_50_ of 8 mg/L and an MIC_90_ of 16 mg/L.^176^ In the same collection that included 101 *S. maltophilia* isolates, the MIC_50/90_ for FEP-ZID was 8/32 mg/L, compared to 32/64 mg/L for cefepime, >64/>64 mg/L for zidebactam and 64 mg/L for CZA.^176^ FEP-ZID also displayed *in vitro* activity against sulbactam-durlobactam (SUL-DUR)-resistant *A. baumannii,* although resistance was due to OXA- and ADC-carbapenemases.^179^ MICs ranged from 32 to >128 mg/L in a collection of 19 MBL-producing *A. baumannii.*^180^ FEP-ZID has relatively low MICs against difficult-to-treat *P. aeruginosa,* demonstrating an MIC_90_ of 8 mg/L against 502 isolates exhibiting this phenotype in the USA, including 8 MBL-producing isolates.^181^

In a neutropenic murine pneumonia model utilizing 8 MBL-producing isolates (2 *E. coli*, 6 *K. pneumoniae)*, FEP-ZID demonstrated 1-log kill at a target of 31% *f*T > MIC for cefepime, which is lower than other cephalosporins.^167^ Similarly, in a neutropenic mouse infection model of *A. baumannii,* the *f*T > MIC target for 1-log kill for cefepime decreased from 38.9% to 15.5% in the presence of zidebactam.^182^ The PK-PD indices for FEP-ZID are complex, as concurrent PBP binding by both drugs demonstrated bacterial killing at concentrations below the MIC for *K. pneumoniae, P. aeruginosa* and *A. baumannii*.^183^ This is observed by (i) cefepime PBP3 binding at low concentrations resulting in cell elongation, (ii) zidebactam PBP2 binding at low concentrations resulting in spheroplast formation and (iii) PBP2 and PBP3 simultaneous inhibition causing a cell morphology that is lysis-prone. This potency was replicated in a lung infection model showing consistent killing against *A. baumannii* even with PK variability.^184^ A higher correlation with PD effects was seen with ‘sub-MIC’ values compared to the more traditional %*f*T > MIC index.^184^ This first-of-its-kind study showed how ‘enhancer’ β-lactamase inhibitors augment antibiotic activity by lowering required β-lactam exposures against gram-negative pathogens.

One of the most important questions facing clinicians is whether FEP-ZID will be an effective treatment option for patients with NDM-producing *E. coli* that harbour PBP3 mutations conferring resistance to ATM-AVI and/or cefiderocol. Watkins *et al.*^185^ performed time-kill assays of cefepime, zidebactam, ATM and FEP-ZID against 6 *E. coli* isolates—three harbouring PBP3 mutations and three wild type. FEP-ZID was synergistic against the wild-type strains, but in the PBP3 mutant strains, FEP-ZID exhibited similar killing to zidebactam monotherapy despite FEP-ZID MICs of ≤0.25/0.25 mg/L, suggesting that activity was due to zidebactam alone. More concerningly, in the isolates with PBP3 mutations, regrowth occurred at concentrations as high as 64/32 mg/L despite initial killing; the regrowth contained mutations conferring resistance to antibiotics targeting PBP2 (i.e. *cysB,* the most common cause of resistance to PBP2 inhibitor mecillinam). A concern for PBP2 inhibition alone is the rapid propensity for resistance development, compensatory PBP1b upregulation and poor outcomes when used as monotherapy, each of which has been reported previously for mecillinam.^186^ Avibactam also binds PBP2 in *E. coli* in a similar manner as zidebactam, leaving the authors to contemplate the potential for cross-resistance with zidebactam in CZA-exposed patients.^173,187^ Interestingly, ATM was synergistic with zidebactam alone in this study, with FEP-ZID in PBP3 mutant *E. coli,* displaying >6_log10_ cfu/mL reductions in all three isolates. ATM-zidebactam was previously determined *in vitro* to be the most potent combination against MBL-producing Enterobacterales.^188^

The clinical efficacy of FEP-ZID was compared to meropenem in adult patients with complicated urinary tract infections in a Phase 3, randomized controlled trial.^189^ Eligible patients were randomized 2:1 to receive FEP-ZID at a dose of 2 g of cefepime, 1 g of zidebactam every 8 hours over a 1 hour infusion or meropenem 1 g every 8 hours over a 30 minute infusion. In preliminary results, clinical cure rates were similar among patients receiving FEP-ZID (96.8%; 272/352) or meropenem (94.9%; 129/177); however, overall success rates were higher for patients receiving FEP-ZID [89% versus 68.4% (absolute difference = 20.6%, 95% CI: 12.3–29.5%)] due to a higher rate of microbiologic eradication. FEP-ZID has also been used successfully in numerous compassionate use cases for adults and paediatrics, including those with invasive infections caused by MBL-producing *Enterobacterales.*^190–195^

Resistance to FEP-ZID (MIC >512 mg/L) has been observed in MBL-producing *Enterobacterales* and *P. aeruginosa.*^185,196,197^ In a collection of carbapenemase-producing *E. coli* from Sweden, including 79 NDM-producing isolates, FEP-ZID MICs ≥32 mg/L were identified in three isolates, each harbouring the V522I substitution in PBP2, a YRIK insertion in PBP3 and NDM-5, suggesting FEP-ZID resistance is multifactorial.^130^ In four isolates with V522I substitutions alone, FEP-ZID MICs ranged from 0.5 to 4 µg/mL, and YRIK/YRIN inserts in PBP3 alone appeared to have no impact on FEP-ZID susceptibility. In a serial-passage experiment of a clinically derived *P. aeruginosa* harbouring the VIM-2 MBL, several resistance-conferring mutations altering the binding affinity of zidebactam for PBP2.^198^ Sastre-Femenia *et al*.^197^ also describe the V516M mutation in PBP2 and its impact on elevating zidebactam MICs observed in *P. aeruginosa.* Resistance to FEP-ZID has been described clinically in a patient with *P. aeruginosa* who received FEP-ZID as salvage therapy as a result of upregulation of the MexAB-OprM efflux system.^199^ In a series of patients with *P. aeruginosa* treated with CZA increased FEP-ZID MICs were seen as a result of altered AmpC expression and upregulation of the MexAB-OprM efflux system.^200^ Thus, the potential for selection of FEP-ZID cross-resistance in MBL-producing *P. aeruginosa* treated either with I-R or CZA regimens may be possible and is largely attributed to increased efflux of the β-lactamase inhibitor agents.

### Aztreonam- and cefepime-nacubactam

Nacubactam (OP0595) is a DBO that inhibits Ambler class A and C serine β-lactamases. Like zidebactam, it has intrinsic antibacterial effects mediated through PBP2 inhibition and functions as a β-lactam enhancer. Nacubactam is being developed in combination with either ATM (ATM-NAC) or cefepime (FEP-NAC). It was previously studied with meropenem as a partner β-lactam, but that combination did not proceed to Phase 3 trials.

Data regarding the *in vitro* activity of ATM-NAC and FEP-NAC against MBL-producing *Enterobacterales* is limited, and the preferred *in vitro* testing method has evolved over time. Breakpoints have not been set for either combination. Nacubactam MICs tend to follow a bimodal distribution against MBL-producing *Enterobacterales.* Against *E. coli* and *Enterobacter* spp., the MICs range from 1 to 8 mg/L; however, against *Providencia* spp., MICs are >32 mg/L, and for *K. pneumoniae* MICs cluster within both groups.^201^ In a global collection of 917 CRE, the MIC_90s_ for ATM-NAC and FEP-NAC were 2 and 4 mg/L, respectively.^202^ Nacubactam MICs were >256 mg/L against Proteeae (*Proteus* spp*., Providencia* spp*., Morganella* spp.) suggesting that an altered PBP2 structure may limit the agent’s antibacterial properties for these species specifically (similar to why zidebactam and imipenem are not potent against these pathogens). Amongst 35 Enterobacterales isolates harbouring both Class B and Class D enzymes, the MIC_50/90_ of ATM-NAC and FEP-NAC was 1/2 mg/L and 2/4 mg/L, respectively.^203^ This is consistent with data suggesting that ATM, a more MBL-stable β-lactam partner, results in more potent *in vitro* activity compared to cefepime- or meropenem-based combinations.^201^ A different study of 64 MBL-producing Enterobacterales from the Swiss National Reference Center for Emerging Antibiotic Resistance (NARA) demonstrated an MIC_50/90_ for ATM-NAC (at a fixed NAC concentration of 4 mg/L) of ≤0.125/8 mg/L.^188^ In this Swiss dataset, ATM-zidebactam (at ZID 8 mg/L) was the most potent combination with an MIC90 ≤0.125 mg/L.

Against 39 clinical MBL-producing *P. aeruginosa* isolates, ATM-NAC inhibited 66.7% (similar to ATM-AVI and ATM alone, which inhibited 69.2% and 53.8%, respectively).^188^ Nacubactam demonstrated relatively poor *in vitro* activity against *P. aeruginosa,* potentially reflecting differences in PBP2 across species.^204^ In a larger database of 1114 consecutive clinical *P. aeruginosa* isolates (including strains with and without MDR phenotypes), the MIC_50/90_ of ATM-NAC and FEP-NAC was 4/16 mg/L and 2/8 mg/L, respectively.^202^ Amongst 196 clinical *A. baumannii* isolates, the MIC_50/90_ of ATM-NAC and FEP-NAC was 32/>64 mg/L and 16/64 mg/L, respectively. In a collection of 53 *S. maltophilia* isolates from Japan, the MIC_50/90_ values were 8/16 mg/L for ATM-NAC and 4/16 mg/L for FEP-NAC.^205^

Although both zidebactam and nacubactam possess dual β-lactamase inhibitory and PBP2-targeting properties, direct comparative studies of PBP2 binding affinity and occupancy across clinically relevant gram-negative pathogens are lacking. Existing evidence suggests species-specific differences in PBP2 engagement that may contribute to variability in antibacterial enhancer effects. Zidebactam appears to have more potent and a broader spectrum of *in vitro* activity compared to nacubactam, although data are limited.^188,206^ This may be best emphasized when both agents are evaluated with the same β-lactam backbone at fixed ratios; however, to our knowledge, such studies have not yet been completed.

As described with zidebactam, the PK-PD profiles of the β-lactam enhancer agents are complex. Given the interdependency of both the β-lactam and the β-lactamase inhibitor to exert antibacterial activity, the exposures of both molecules in the blood at any given time are important to understand. The concept of an instantaneous MIC (MICi) was introduced with the development of imipenem-relebactam to mathematically model and simulate how β-lactamase inhibitor concentration changes the MIC of the β-lactam over time.^207^ Essentially, the MIC is not a static number but rather changes over time—which is likely true for β-lactam monotherapy as well.^208^ This %fT > MICi modelling supported dosing of ATM-NAC and FEP-NAC in Phase 3 trials.

The Integral-1 phase 3, randomized, controlled trial evaluated efficacy of ATM-NAC and FEP-NAC in adult patients with complicated urinary tract infections.^209^ Four hundred thirty-one patients were randomly assigned (2:1:1) to receive FEP-NAC 2 g/1 g every 8 hours, ATM-NAC 2 g/1 g every 8 hours or imipenem 1 g every 8 hours, each over a 60 minute infusion. Of note, nacubactam does require renal dose adjustment and is efficiently cleared during haemodialysis.^210^ The primary endpoint of composite clinical and microbiologic success was achieved in 82.2% (176/214), 72.3% (81/112) and 61% (64/105) of patients, respectively. Compared to treatment with imipenem, both agents met criteria for non-inferiority, whereas the combination of FEP-NAC met criteria for superiority, which was driven by microbiological eradication. A subsequent trial (Integral-2) randomizing patients to ATM-NAC, FEP-NAC or BAT for CRE infections has completed enrolment, but results have not yet been published (NCT05905055). Investigators randomized either nacubactam combination versus BAT in a 2:1 ratio based on known or suspected mechanism of carbapenem resistance (i.e. FEP-NAC if serine carbapenemase or non-carbapenemase resistance, ATM-NAC if MBL).

Clinical resistance development to nacubactam-based antibiotics has not been described due to limited use and no real-world data available. However, intrinsic and acquired resistance exist at baseline for all antibiotics with certain pathogens, and it is anticipated that FEP-NAC will share resistance mechanisms with FEP-ZID.^201^

### Sulbactam-durlobactam

Durlobactam is a DBO β-lactamase inhibitor that is structurally similar to AVI, but optimized to inhibit class D carbapenemases, including OXA-23/24 that are common in carbapenem-resistant *A. baumannii.*^211^SUL-DUR received FDA approval in 2023 as a fixed combination for the treatment of carbapenem-resistant *A. baumannii* infections. Like other DBOs, durlobactam cannot inhibit MBLs; however, it demonstrates intrinsic activity through direct PBP2 inhibition in Enterobacterales. There are no published PK-PD assessments evaluating the optimal exposures of durlobactam as a dual-functioning β-lactamase inhibitor and enhancer for Enterobacterales.

Aitken *et al*.^212^ explored the *in vitro* activity of durlobactam against NDM-5-producing *E. coli* with PBP3 insertions from five unique patients at two centres in the USA. The authors hypothesized that activity would be due to the PBP2 affinity of durlobactam and that durlobactam is not hydrolysed by MBLs. Indeed, durlobactam MICs ranged from 0.5 to 2 mg/L (compared to ATM-AVI 2–16 mg/L and cefiderocol 1 to >32 mg/L). Le Terrier *et al*.^213^ then evaluated durlobactam alone or in combination against 110 consecutive, nonduplicate NDM-producing *E. coli* from the Swiss NARA)from January 2023 to May 2024. Most were NDM-5 (*N* = 87). Durlobactam (fixed at 4 mg/L) alone inhibited 100% of isolates with an MIC_90_ of 1 mg/L. Durlobactam does not appreciably inhibit PBPs of *P. aeruginosa* or *S. maltophilia,* but it will inhibit Class A, C and D enzymes found in these pathogens.

SUL-DUR has been used to treat 17 patients with polymicrobial infections including carbapenem-resistant *A. baumannii* plus MBL-producing Enterobacterales and/or carbapenem-resistant *P. aeruginosa.*^214,215^ Yang *et al.*^215^ describe success of using ATM plus SUL-DUR to treat a pulmonary co-infection of MBL-producing Enterobacterales and carbapenem-resistant *A. baumannii.* As with all antibiotics, potential for treatment-emergent resistance exists. A laboratory-derived mutant strain of NDM-producing *E. coli* displayed resistance via a point mutation in the *mrdA* gene, which resulted in a PBP2 mutation.^129^

## β-Lactam/MBL inhibitor combinations

### Cefepime-taniborbactam

Developing an MBL inhibitor is challenging because the overall fold of MBLs are like many metal-containing enzymes across all three domains of life. This lack of selectivity to bacterial enzymes can result in off-target effects, leading to more toxicity relative to inhibitors of serine β-lactamases that are unique to bacteria. There is also no mechanistic covalent intermediate like the one created during serine β-lactamase hydrolysis, making an inhibitor harder to design. As outlined above, durlobactam, zidebactam and nacubactam do not inhibit MBLs. Lisa *et al.*^19^ noted that some MBLs share a reaction mechanism and were able to characterize the Michaelis complex formed by mono-Zn(II) enzymes and identify all intermediate species, enabling them to propose a chemical mechanism for mono- and binuclear MβLs and suggest a strategy for drug design. This mechanism is also characterized by an initial tetrahedral transition state resembling the one present in serine β-lactamase-mediated catalysis.

Taniborbactam (VNRX-5133) is an investigational bicyclic boronic acid β-lactamase inhibitor that was explored with cefepime as the parent β-lactam. It was superior to meropenem in a Phase III trial (CERTAIN-1) of patients with complicated urinary tract infection, but the trial enrolled few patients with MBLs.^216^

Similar to other boronate-based inhibitors, taniborbactam does not have its own antibacterial activity via PBP binding and functions only as a β-lactamase inhibitor.^217^ Crystallographic studies show that taniborbactam binds to both serine β-lactamases (SBLs) and MBLs in an sp^2^-hybridized state that mimics the β-lactam scaffold.^218^ Once positioned in the active site, they react with either the nucleophilic serine (in SBLs) or the Zn(II)-bridged hydroxide (in MBLs) to form a tightly bound sp^3^ complex resembling the tetrahedral transition state of β-lactam hydrolysis. Taniborbactam inhibits most NDM and VIM variants effectively, but not IMP-like enzymes or most B2 and B3 MBLs, including L1 from *S. maltophilia*.

In the GEARS global surveillance programme, cefepime-taniborbactam inhibited 95% of 42 VIM-positive and 76% of 364 NDM-positive Enterobacterales isolates at an MIC ≤16 mg/L.^219^ In this same database, cefepime-taniborbactam inhibited 82% of 271 VIM-positive *P. aeruginosa* at an MIC ≤16 mg/L. In France, cefepime-taniborbactam inhibited 76% of NDM-producing Enterobacterales at an MIC ≤16 mg/L.^220^ MICs were lower for NDM-1-producing isolates compared to those harbouring NDM-5. Resistance was more common amongst *E. coli* species compared to *K. pneumoniae*.

Resistance to taniborbactam in isolates harbouring NDM-9 was discovered during its Phase 3 development.^221^ Drusin *et al.*^11^ investigated the molecular basis for this using docking simulations. In susceptible enzymes such as NDM-1 and VIM-2, the active-site surface contains a negatively charged patch around Glu149 and Asp236 that forms favourable electrostatic interactions with the positively charged n-(2-aminoethyl)cyclohexylamine moiety of taniborbactam. Substitutions at these positions—Glu149Lys in NDM-9 and VIM-83 or Asp236Tyr in NDM-30—reduce the negative potential of this region, disrupting and weakening key electrostatic interactions and diminishing inhibitor binding.^221,222^ Similarly, a positively charged surface conserved in IMP enzymes precludes a favourable binding of taniborbactam, accounting for the lack of inhibition. Additionally, cefepime-taniborbactam resistance was seen in both *P. aeruginosa* and Enterobacterales resulting from accumulation of multiple porin alterations, overexpression of efflux pumps and alterations in PBP3.^223^ Cefepime-taniborbactam had limited *in vitro* activity against ATM-AVI -resistant *E. coli* harbouring PBP3 insertions and expressing CMY-42.^177^

The new drug application for cefepime-taniborbactam was rejected by the FDA in early 2024 due to a request for ‘additional chemistry, manufacturing and controls’ information. To our knowledge, there is no intention to further develop this combination at this time.

### Cefiderocol-xeruborbactam

Xeruborbactam (QPX7728) is another bicyclic boronate in clinical development with a broad, potent inhibitory spectrum. In enzyme kinetic studies, xeruborbactam inhibits most known serine β-lactamases and MBLs including Class D OXA type enzymes, NDM, VIM and most IMP variants (although with relatively less potency compared to other MBLs), but it does not inhibit the L1 present in *S. maltophilia.*^224^ Unlike taniborbactam, xeruborbactam can inhibit NDM-9 since it lacks a bulky, positively charged side chain and it therefore avoids the electrostatic repulsion that led taniborbactam to poor inhibition.^225^

Xeruborbactam does possess some intrinsic antibacterial activity via PBP inhibition.^206^ This inhibition is generally less potent than that of the DBO class and hypothesized to be more aligned with PBP inhibition seen with sulbactam (PBP1 and PBP3 in *A. baumannii*) and AVI (PBP2 in *E. coli*, *Haemophilus influenzae* and *P. aeruginosa*). In a collection of 1028 Enterobacterales, xeruborbactam alone demonstrated an MIC_90_ of 16 mg/L against *E. coli,* 32 mg/L against *Klebsiella oxytoca* and *K. pneumoniae* and 64 mg/L against *Enterobacter cloacae.* However, 94% of 506 clinical isolates of *P. aeruginosa* displayed xeruborbactam MICs of ≥64 mg/L.

A unique aspect of xeruborbactam relative to other β-lactamase inhibitors is availability of a sodium ester prodrug with reasonable bioavailability allowing for potential development as part of an oral combination product.^226^ Thus, xeruborbactam has entered early-stage clinical development both as a co-formulated oral product with ceftibuten (NCT06157242) and as an intravenous product co-formulated with cefiderocol (NCT06157242).^227^

Le Terrier *et al.*^228^ evaluated the in vitro activity of the meropenem-xeruborbactam combination against a broad panel of IMP-producing gram-negative isolates to identify potential resistance mechanisms. In *P. aeruginosa*, the combination showed no meaningful MIC reduction compared with meropenem alone, likely reflecting efflux-mediated limitation of xeruborbactam activity in this species. In Enterobacterales producing IMP-6, IMP-14, IMP-26 or IMP-34, less than a 2-fold MIC decrease was observed, a pattern reproduced when these enzymes were expressed in *E. coli*. Biochemical assays confirmed minimal inhibition of IMP-6, IMP-10, IMP-14 and IMP-26 by xeruborbactam, with IC_50_ values near 100 µM.

Structural modelling data suggest that a Ser262Gly substitution in IMP-6 alters the electronic environment of the active site by disrupting the interaction between Ser262 and Cys221, thereby reducing xeruborbactam binding.^228^ This substitution may also perturb a hydrogen-bonding interaction at the base of loop L3, shifting its conformation and affecting substrate recognition. Additionally, the Val67Phe substitution in L3, present in IMP-10 and IMP-26, introduces steric hindrance that may further impede proper positioning of xeruborbactam within the active site.

Like other β-lactamase inhibitors, the activity of xeruborbactam-containing combinations is partially dependent on non-β-lactamase-mediated mechanisms of resistance in the organism. For example, OmpF/OmpK35 and/or OmpF/OmpK36 have previously been demonstrated to restrict meropenem and xeruborbactam entry and have been associated with the increase in meropenem-xeruborbactam MICs in *K. pneumoniae*, as have efflux pumps and porin alterations in *P. aeruginosa* and *A. baumannii.*^229^ When combined with cefiderocol, xeruborbactam restores activity against Enterobacterales harbouring NDM, but this activity may be abrogated in the presence of concomitant alterations in iron uptake systems or PBP3 insertions altering the efficacy of cefiderocol.^229,230^

### Conclusions

MBLs represent a dynamic and evolving global threat, with regional differences in enzyme prevalence increasingly blurred by international dissemination. The complex resistance profiles of MBL-producing pathogens often leave clinicians with few reliable treatment options, underscoring the urgent need for enhanced surveillance, novel therapeutics and individualized management strategies to improve patient outcomes. Despite the availability of new antibiotic options for MBL infections, significant clinical challenges that require rapid diagnostics, optimized antimicrobial therapy and coordinated stewardship and infection prevention efforts remain.

## Supplementary Material

dlag221_Supplementary_Data

## Transparency declarations

E.K.M. has served on an advisory board for Melinta (now Cormedix), GSK, Basilea, Biomerieux and Beckman Coulter. She has received honoraria from Biomerieux, GSK and Cormedix for speaking at ESCMID Global and the Sepsis Alliance. A.J.V. had a research contract with Shionogi and has received honoraria from Pfizer as a speaker. R.K.S. has received investigator-initiated research grants from AbbVie, Melinta, Merck, Innoviva and Shionogi. He has also served as a consultant for AbbVie, Merck, bioMérieux, Roche, Qpex, GlaxoSmithKline and Informuta. S.L.A. has served as a consultant for Melinta, Merck, Shionogi, Wockhardt, Alembic, Innoviva, Meitheal, bioVersys, bioMérieux, Roche and Guidepoint Global Advisors and has received honoraria for non-CME medical education for Shionogi. J.M.P. has received investigator-initiated research grants from Merck, Shionogi, Abbvie, Cor-Medix and Innoviva and has served as a consultant for GlaxoSmithKline, Wockhardt, Shionogi and Q-linea. E.L.H. and M.F.M. have nothing to disclose.
