# Supplementary material for: The global epidemiology and treatment of metallo-β-lactamase-producing gram-negative pathogens
Source: JAC Antimicrob Resist. 2026 Oct 14;8(5):dlag221. doi: 10.1093/jacamr/dlag221 (PMC13662407; doi:10.1093/jacamr/dlag221)
Supplement: Supplementary file 1 [file dlag221_supplementary_data.docx]

**Supplementary Material**

**The global epidemiology and treatment of metallo-β-lactamase-producing gram-negative pathogens**

**Construction of epidemiological maps for IMP, VIM, and NDM.**

The global prevalence maps (**Figure 1**) were generated by updating previously published maps of metallo‑β‑lactamase distribution from two prior reviews authored by Mojica et al. The 2016 review included an exhaustive PubMed search of all reports of IMP, VIM, and NDM enzymes across all recognized countries worldwide up to 2016, and the 2022 review provided an updated synthesis focused on NDM.

For the present manuscript, these earlier maps served as the baseline framework. We updated them using peer‑reviewed literature and surveillance reports published between 2015 and 2025, including systematic reviews and epidemiological summaries and official global and national AMR surveillance reports (see reference list below). All epidemiological papers cited throughout the manuscript were also incorporated.

For each country, we extracted whether IMP, VIM, or NDM had been reported; the frequency and recency of reports; whether detection was sporadic or sustained; and whether endemic circulation was documented. Countries were assigned to prevalence tiers (high/widespread, moderate/potential for widespread dissemination, low/emerging, or no reliable data) based on qualitative synthesis of these sources. Maps were created using Datawrapper.

**Figure 1.** Global epidemiology of IMP (A), VIM (B), and NDM (C) metallo‑β‑lactamases across gram‑negative species. Prevalence was estimated based on reports in published literature; additional methodological details on data sources and map construction are provided in the Supplementary Material. Countries in red indicate widespread dissemination; countries in orange indicate potential for widespread dissemination; and countries in green indicate isolated reports or early emergence of the enzyme(s). Countries shown in gray represent regions where no reliable data are available. Figure created with Datawrapper (<https://app.datawrapper.de>).

**References for Figure 1**

Mojica MF, Bonomo RA, Fast W. B1-Metallo-β-Lactamases: Where Do We Stand? Curr Drug Targets. 2016;17(9):1029-50. doi: 10.2174/1389450116666151001105622. PMID: 26424398; PMCID: PMC4814356.

Mojica MF, Rossi MA, Vila AJ, Bonomo RA. The urgent need for metallo-β-lactamase inhibitors: an unattended global threat. Lancet Infect Dis. 2022 Jan;22(1):e28-e34. doi: 10.1016/S1473-3099(20)30868-9. Epub 2021 Jul 8. PMID: 34246322; PMCID: PMC8266270.

Bonnin, R. A. *et al.* Genetic diversity, biochemical properties, epidemiology, and detection methods of IMP- and VIM-type carbapenemases. *Clin. Microbiol. Rev.* **38**, e0012923 (2025).

Pongchaikul, P. & Mongkolsuk, P. Comprehensive Analysis of Imipenemase (IMP)-Type Metallo-β-Lactamase: A Global Distribution Threatening Asia. *Antibiotics* **11**, 236 (2022).

Kanj, S. S., Kantecki, M., Arhin, F. F. & Gheorghe, M. Epidemiology and outcomes associated with MBL-producing Enterobacterales: A systematic literature review. *Int. J. Antimicrob. Agents* **65**, 107449 (2025).

Falagas, M. E., Kontogiannis, D. S., Zidrou, M., Filippou, C. & Tansarli, G. S. Global Epidemiology and Antimicrobial Resistance of Metallo-β-Lactamase (MBL)-Producing Acinetobacter Clinical Isolates: A Systematic Review. *Pathogens* **14**, 557 (2025).

Aslan, A. T. & Paterson, D. L. Epidemiology and clinical significance of carbapenemases in Australia: a narrative review. *Intern. Med. J.* **54**, 535–544 (2024).

Pillonetto, M. *et al.* Carbapenemases producing gram-negative bacteria surveillance in Latin America and the caribbean: a retrospective observational study from 2015 to 2020. *Lancet Reg. Health Am.* **49**, 101185 (2025).

Grossman, J. M. & Thompson, D. K. Clinically Significant Carbapenemases in Gram-Negative Pathogens: Molecular Diversity and Advances in β-Lactamase Inhibitor Therapy. *Antibiotics* **15**, 413 (2026).

Dos Santos CS, Silva MJA, Dos Santos PAS, de Miranda EVC, Duarte ABT, Aires CAM, Lima LNGC, Brasiliense DM, Souza CO, Lima KVB, Rodrigues YC. Nationwide Burden of Metallo-β-Lactamase Genes in Brazilian Clinical *Klebsiella pneumoniae* Isolates: A Systematic Review and Meta-Analysis. Antibiotics (Basel). 2025 Sep 19;14(9):951. doi: 10.3390/antibiotics14090951. PMID: 41009929; PMCID: PMC12466462.

Sadeghi H, Khanjani S, Aslanimehr M, Aria N, Maleki MR, Nikkhahi F, Gheibi N, Marashi SMA, Khoei SG. Encoding Genes of Metallo β-Lactamases (*IMP*, *NDM*, and *VIM*) in *Klebsiella Pneumoniae* in Iran: A Systematic Review and Meta-Analysis. Health Sci Rep. 2025 Jun 18;8(6):e70927. doi: 10.1002/hsr2.70927. PMID: 40535515; PMCID: PMC12174657.

Sisay A, Kumie G, Gashaw Y, Nigatie M, Gebray HM, Reta MA. Prevalence of genes encoding carbapenem-resistance in Klebsiella pneumoniae recovered from clinical samples in Africa: systematic review and meta-analysis. BMC Infect Dis. 2025 Apr 18;25(1):556. doi: 10.1186/s12879-025-10959-7. PMID: 40251495; PMCID: PMC12007206.

Chatzidimitriou M, Kavvada A, Kavvadas D, Kyriazidi MA, Eleftheriadis K, Varlamis S, Papaliagkas V, Mitka S. Carbapenem-resistant Klebsiella pneumoniae in the Balkans: Clonal distribution and associated resistance determinants. Acta Microbiol Immunol Hung. 2024 Feb 21;71(1):10-24. doi: 10.1556/030.2024.02230. PMID: 38381174.

Chelaru EC, Muntean AA, Hogea MO, Muntean MM, Popa MI, Popa GL. The Importance of Carbapenemase-Producing Enterobacterales in African Countries: Evolution and Current Burden. Antibiotics (Basel). 2024 Mar 24;13(4):295. doi: 10.3390/antibiotics13040295. PMID: 38666971; PMCID: PMC11047529.

European Centre for Disease Prevention and Control. Increase in Escherichia coli isolates carrying blaNDM-5 in the European Union/European Economic Area, 2012–2022. Stockholm: ECDC; 2023

World Health Organization. Global Antimicrobial Resistance and Use Surveillance System (GLASS) Report 2025. Geneva: World Health Organization; 2025.

European Centre for Disease Prevention and Control (ECDC). Antimicrobial resistance in the EU/EEA (EARS‑Net) – Annual Epidemiological Report 2024. Stockholm: ECDC; 2025

Instituto Nacional de Salud de Colombia. Memorias Reunion de expertos: Carga de la resistencia antimicrobiana en Colombia. Retrieved September 1, 2025 from <https://www.ins.gov.co/Direcciones/ONS/SiteAssets/memoriasRAM%20resistencia%20antimicrobiana.pdf>

Administracion Nacional de Laboratorios e Institutos de Salud Dr. Carlos Malbran. Vigilancia de la Resistancia Antimicrobiana. Retrieved September 1, 2025 from <https://www.argentina.gob.ar/vigilancia-de-la-resistencia-antimicrobiana>

Agencia Nacional de Vigilancia Sanitaria (ANVISA). Boletim epidemiologico - Microrganismos resistentes aos carbapenêmicos e sua distribuição no Brasil, 2015 a 2022. Retrieved September 1, 2025 from <https://www.gov.br/saude/pt-br/centrais-de-conteudo/publicacoes/boletins/epidemiologicos/edicoes/2024/boletim-epidem-vol-55-n-2/view>

Salud - Sitio Oficial de la Republica Oriental del Uruguay. Periodo 2019 - 2023: Numero de organismos productores de carbapenemasas. Retrieved September 1, 2025 from <https://www.gub.uy/ministerio-salud-publica/institucional/informacion-gestion/informes-sobre-solicitudes-acceso-informacion-publica-2024-93>
